# Proteoglycan 4 predicts tribological properties of repaired cartilage tissue

**DOI:** 10.7150/thno.39386

**Published:** 2020-01-22

**Authors:** Zhiguang Qiao, Mei Xin, Ling Wang, Huiwu Li, Chengtao Wang, Liao Wang, Tingting Tang, Bangshang Zhu, Gang Huang, You Wang, Minghao Zheng, Kerong Dai

**Affiliations:** 1 Shanghai Key Laboratory of Orthopaedic Implants, Department of Orthopaedics, Ninth People's Hospital, School of Medicine, Shanghai Jiao Tong University, Shanghai 200011, China.; 2 Department of Nuclear Medicine, Renji Hospital, School of Medicine, Shanghai Jiao Tong University, Shanghai 200127, China.; 3 State Key Laboratory for Manufacturing System Engineering, School of Mechanical Engineering, Xi'an Jiao Tong University, Xi'an 710054, China.; 4 School of Biomedical Engineering, Shanghai Jiao Tong University, Shanghai 200240, China.; 5 Engineering Research Center of Digital Medicine, Ministry of Education, Shanghai 200030, China.; 6 Center of Analysis and Test, Shanghai Jiao Tong University, Shanghai 200240, China.; 7 Shanghai University of Medicine and Health sciences, Shanghai 201318, China.; 8 Department of Bone and Joint Surgery, Renji Hospital, School of Medicine, Shanghai Jiao Tong University, Shanghai 200127, China.; 9 Centre for Orthopaedic Research, School of Surgery, The University of Western Australia, Crawley 6009, Australia.

**Keywords:** Proteoglycan 4, cartilage regeneration, biotribology, wear resistance, bone marrow enrichment.

## Abstract

**Purpose**: One of the essential requirements in maintaining the normal joint motor function is the perfect tribological property of the articular cartilage. Many cartilage regeneration strategies have been developed for treatment in early stages of osteoarthritis, but there is little information on how repaired articular cartilage regains durability. The identification of biomarkers that can predict wear resistant property is critical to advancing the success of cartilage regeneration therapies. Proteoglycan 4 (PRG4) is a macromolecule distributing on the chondrocyte surface that contributes to lubrication. In this study, we investigate if PRG4 expression is associated with tribological properties of regenerated cartilage, and is able to predict its wear resistant status.

**Methods**: Two different strategies including bone marrow enrichment plus microfracture (B/BME-MFX) and microfracture alone (B-MFX) of cartilage repair in sheep were used. PRG4 expression and a series of tribological parameters on regenerated cartilage were rigorously examined and compared.

**Results**: Highly and continuously expression of PRG4 in regenerated cartilage surface was negatively correlated with each tribological parameter (*P*<0.0001, respectively). Multivariate analysis showed that PRG4 expression was the key predictor that contributed to the promotion of cartilage wear resistance.

**Conclusion**: Higher PRG4 expression in regenerated cartilage is significantly associated with wear resistance improvement. PRG4 may be useful for predicting the wear resistant status of regenerated cartilage and determining the optimal cartilage repair strategy.

## Introduction

Articular cartilage defects that commonly occur due to trauma can result in the increase of wear and tear, loss of joint function and the reduction of quality of life 1, 2. Because cartilage does not have capability of self-healing 3, numerous biological therapeutic approaches, including use of autologous chondrocytes, stem cells and bone marrow stimulation, have been studied to attempt to preserve existing, and to repair damaged cartilage tissue 4-8. While ideal repaired cartilage tissue is to fulfill its ability to bear pressure, maintain elasticity and provide a smooth motion pattern that resists wear and tear, the current evaluations on the cartilage repair methods have been often limited to conventional histological assessments or biomechanical tests 9-12. Histological assessment has been a fundamental criterion evaluation of cartilage repair. From International Cartilage Repair Society (ICRS) I to ICRS II scoring system, in order to accurately reflect the genuine functional property of the regenerative cartilage as far as possible, previous researchers have committed to constantly revise and improve the histological criteria in the clinical practice 13-15. However, little is known on whether the repair cartilage can fulfill the biological function and provide sufficient wear resistance during joint movement. Most of the studies only focus on the surface friction coefficient 16-18 which does not provide the biological durability of cartilage.

The tribological property of cartilage is essential in supporting and guaranteeing normal joint movements during the spectrum of life. The long-term integrity of an articulation depends on the nourishment of its cartilage component and the protection of the cartilage surface from the inevitable mechanical abrasion 19. Proteoglycan 4 (PRG4) 20, a 345 KDa proteoglycan distributed along the surface layer of cartilage, contributes greatly to diminishing the friction coefficient between the interfaces, and thus protecting the cartilage surface from the friction-induced wear 21. To date, however, the role of PRG4 on wear resistance of regenerated cartilage, was not clear.

In this study we hypothesize that the quality of cartilage tissues regenerated by biological approaches depends on its tribological properties. Using two different cartilage defect models in sheep, we have therefore designed a series of tests to rigorously validate if PRG4 can be used as a biomarker to predict tribological properties of repaired cartilage tissue (Figure S1). We showed that PRG4 is associated with the tribological performance of regenerative tissues and PRG4 can be a useful biomarker to predict the wear resistant property of the cartilage repairs.

## Materials and Methods

### Cartilage defect preparation

A total of 96 male Shag Mongolia sheep at 15 months of age were initially enrolled in this study. Joint diseases were excluded by preoperative radiological examination. The average animal weight was 40.0 ± 0.7 kg. All the experiments with live animals were approved by the Ethics Committee of Shanghai Jiao Tong University School of medicine and were conducted in compliance with the Guide for Care of Laboratory Animals as detailed by the National Ministry of Science.

The animals were sedated with 0.1 mg fentanyl prior to surgery, administrated with thiopentone (25 mg/kg), ventilated with O_2_ in N_2_O by volume control and the whole anaesthesia was maintained with 1.5-2 vol% isoflurane. The stifle joint of the right hind leg was sterilely opened by an anteromedial approach. The incision was extended into the joint capsule and the patella was subluxated laterally. Cartilage defect was outlined by a custom-designed 7 mm punch (Surgical instruments factory Shanghai Co., LTD) in the medial trochlear groove of the distal femur, when the cartilage surface was pressed vertically until a greater resistance encountered from the subchondral bone, then the full-layer cartilage including calcified cartilage zone was removed. No bleeding from the subchondral bone was observed during the surgery.

### Treatment groups

Cartilage defects were treated using microfracture (MFX) followed by filling the full-thickness cartilage defect with Bio-Gide^®^ collagen membrane (B). For one group, the Bio-Gide^®^ collagen scaffold was seeded with cells obtained from bone marrow enrichment (BME) before insertion and was denoted as B/BME-MFX (n=34, Figure S2A); while for the other, pure Bio-Gide^®^ collagen scaffold was inserted after performing microfracture and was denoted as B-MFX (n=32). Operation defect alone (Defect, n=18) without artificial repair was defined as control for surgical intervention and cartilage repair (Figure S2B). Sham operation group (Sham, n=12) was especially established as control for wear resistance evaluation. All these sheep were received MRI scan at the 6th, 12th and 24th weeks.

### Gross observation and histological analysis

After the surgery, additional three sheep from B/BME-MFX, B-MFX and Defect groups were randomly selected and sacrificed with euthanasia for gross observation at the 6th and 12th weeks, and the others [B/BME-MFX (n=28), B-MFX (n=26), Defect (n=12), Sham (n=12)] were left until the final day of the 24th week for ICRS II scoring, histological analysis and the subsequent wear test. Gross examination and histological evaluation were performed blindly by two independent pathologists.

Centered by cartilage defect repair area (7 mm in diameter), specimen blocks (10×10 mm) from the distal femora of different groups were firstly osteotomized. The specimen was segmented for histological analysis at about 2 mm from the boundary of the defect area by a bone saw, and the remaining part was left for the subsequent wear test. During the histological preparation procedure, samples were formalin-fixed, decalcified in 0.5 M ethylene diamine tetraacetic acid (EDTA) solution, paraffin-embedded (FFPE) and sectioned into 5 μm thick slides longitudinally. Both the cationic dyes, Alcian blue (AB) and Safranin O-Fast Green (SO/FG), were used to detect proteoglycans, especially glycosaminoglycans in the cartilage matrix. The large molecule size of the chemical components in the Alcian blue were suitable for the qualitative assay, while Safranin O was more fit for the specific quantitative determination of the cartilage polyanions. Immunohistochemistry (IHC) staining was performed using the primary antibodies: Collagen II (COL II) (1:100, ab34712, Abcam, USA), SOX-9 (1:250, sc166505, Santa-Cruz, USA), and PRG4/Lubricin/SZP (1:200, ab28484, Abcam). Secondary antibody staining and peroxidase detection were performed with an EnVision™ Detection Systems (K5007, Dako, Glostrup, Denmark) according to manufacturer's instructions.

The staining results of SO/FG and IHC were ranked blindly and semi-quantitated by two independent investigators, using a grading system based on the percentage of positive staining cells and the intensity of staining from at least five fields under the microscope 22-24. The density score ranged from 0 to 4 (0, no positive cells; 1, <25%; 2, 25%-50%; 3, 50%-75%; 4, positive cells > 75%), and the intensity score was also measured on a scale of 0 to 3 (0, none; 1, weak; 2, moderate; 3, strong). The multiplication of the two scores yielded the final assessment of the protein expression quantity, with grading from 0 to 12: negative (score 0-1), weak (score 2-3), moderate (score 4-6), strong (score 9-12). “PRG4 surface continuity” ranged from 0 (none) to 100% (intact), referring to the percentage that positive PRG4 staining “line” expressed along the superficial zone of the cartilage.

### Sample preparation for the wear test

Samples from different groups [B/BME-MFX (n=28), B-MFX (n=26), Sham (n=12)] were prepared for wear test. Reciprocal grinding plugs (2 mm in diameter), vertically containing a 2-mm cartilage at the upper edge and an 8-mm osseous tissue beneath, were respectively harvested from the medial femoral condyle of the contralateral intact leg of each sheep. The plugs were fixed onto holders via the osseous end, and the cartilage portion was kept outward for the wear test. A self-designed laser alignment device was used to keep the cartilage surface parallel to the direction of the reciprocating motion during the sample installation.

### *Ex vivo* reciprocating wear test

The *ex vivo* million-cycle reciprocating cartilage-on-cartilage wear tests were conducted following the previously established methods 18, 25-27. The experiments were carried out at 22℃ in room temperature, an air humidity of 70%, and an air cleanness of class 1000. The core experiment settings of the wear test included the grinding frequency (96 cycles per minute) and the maximum reciprocating cycle (one million times), were designed based on the fast walk exercise load of a unilateral knee in two years 28.10 mL of phosphate buffered saline (PBS, 0.15M NaCl solution, pH 7.4, HyClone, Thermo scientific, USA) was used as the lubricating fluid. Considering the natural evaporation, the total volume of the lubricating fluid was maintained at 10 mL by adding distilled water during the whole test automatically. The lubricating fluid was collected and completely replaced by fresh PBS every 200,000-wear cycle.

*In vivo*, the average pressure on the cartilage surfaces of the knee has been measured to be 0.71 MPa with a peak pressure at about 2.1 MPa 29. An average contact pressure of 1.2 MPa was chosen in the wear test according to the physiological condition. As the reciprocating locomotion went on, the wear point on the tested cartilage repair surface shifted along, so as the local bearing pressure, periodically ranging from 0 to 1.2 MPa. Since the small size of the samples and the elasticity of the cartilage, a flat-on-flat contact mode was assumed. Considering each pair of cartilage samples contain 2 mm- and 7 mm-diameter plugs, the reciprocating sliding amplitude was set at 3 mm in order to ensure a full contact between the cartilage pairs during the wear test.

In order to compensate for any possible loss of cartilage components during the agitation in the lubricating fluid, blank control experiments under the identical test conditions, with no load or contact between the specimens were also conducted. As illustrated in Figure 3A and B, a pair of frozen specimens, was thawed in PBS at room temperature and fixed to the loading clamp. Fresh PBS was then placed in the sample pool and the reciprocating sliding program was set to start following the different loading options (“blank control” at 0 MPa, and wear test samples at 1.2 MPa). The real-time coefficient of friction (COF) was recorded during the whole test. When the experiment ended, the cartilage specimens were placed in the fresh PBS and kept moist prior to refreezing for the future studies.

### Surface morphology and roughness evaluations

According to the previous studies 30, contour measurements of the cartilage surface were taken both before and after the wear test by a 3D optical profiler (Bruker Contour GTI, Germany). The VSI mode and 20× amplified objective lens was used, and the roughness parameters (Ra, Rq and Rt) of each specimen were measured. These parameters respectively represent the arithmetic mean (Ra), mean square root (Rq) and maximum amplitude (Rt) of the roughness.

### micro-CT based volume loss evaluation

The cylinder plugs were scanned using a high-resolution micro-CT system (μCT80, Scanco Medical, Switzerland), pre and post the wear test, following established methods 31. The measurements were performed under saturated humidity conditions. Afterwards, the image reconstruction of the cartilage layer was processed by Mimics 11.0 software (Materialise, Belgian) and the cartilage volume of each specimen was analyzed by Geomagic Studio 2013 system (Geomagic, USA). The amount of the cartilage volume loss (△V) generated by one million grinding cycles was calculated.

### Weight loss evaluation

The lubricating fluid from every 200,000 wear cycle was collected into a sterile 50-mL Blue Cap tube (Corning, USA). Each empty tube was individually weighed in advance. Then the sample pool was rinsed by sterile distilled water for 5 times, and every drop of the cleanout fluid was also collected into the same tube. After thoroughly mixing, half weight of the mixture was moved into a new tube for the other experiments, and the rest of the contents were weighed again, together with the tube, after the complete freeze-drying procedure (LABOCONCO FreeZone 6 Plus, USA). The weight loss from the surface of the cartilage pairs (△W) was calculated according to the formula ①.

△W = (W'- W_T_ - W_PBS_) × 2 ①

In this formula, W' is the total weight of the freeze-dried tube, W_T_ is the weight of the same empty tube, W_PBS_ represents the weight of the solute contained in the 5 mL-PBS solution. Sartorius Cubris® MSA Analytical Balance System (Germany) was adopted in all the weighing steps (maximal precision=10 ug).

### *In vitro* chondrogenesis pellet culture

MFX bone marrow was harvested from the bone marrow blood exudation by drilling on the subchondral bone after the removal of full-layer articular cartilage in the defect repair area. According to our previous study of BME bone marrow collection 32, approximately 30 mL of bone marrow was harvested from the bilateral iliac crests of the animals using a 12-gauge beveled needle and a 20-mL syringe rinsed by the heparinized saline beforehand. A 10-min centrifugation at 1500 rpm forced the different elements in the marrow separate by the specific density. The middle layer containing the mesenchymal stem cells (MSC) and other nucleated cells was collected. The final volume of BME was about 3-4 mL. A 500 μL of the BME or MFX bone marrows was separately collected and centrifuged at a speed of 300g for 15 min using a 4℃ centrifuge (HEART LABOFUGE 400 R, Thermo Scientific, USA). The supernatant was removed, and the chondrogenesis inducing medium containing transforming growth factor-β3 (TGF-β3) (R&D Systems, Inc. USA), recombinant human insulin, sodium pyruvate, dexamethasone, levorotatory vitamin C, transferrin, selenite, bovine serum albumin, and linoleic acid (Sigma, USA) was added into the sediment. Then the samples were cultured in a 37℃ incubator. In addition, about 10^7^ MSCs were collected by the plate culture of 5 mL microfracture bone marrow. These cells were also chondrogenesis pellet cultured as the positive control. After a 28-day incubation, the pellets were harvested, formalin-fixed, paraffin-embedded and sectioned into slides for the SO/FG, and IHC studies to detect the production of PRG4 and COL II were performed. All the experiments were repeated at least three times.

### Statistical analysis

Statistical analyses were conducted using the SPSS 17.0 (SPSS, Chicago, IL, USA) and SIMCA 14.0 (Umetrics, Umeå, Sweden) statistical software package. One-way ANOVA and Student's *t* test were performed to compare COF, volume loss, weight loss, surface roughness, compressive modulus, and surface elastic modulus among the different groups. Kruskal-Wallis test and Mann-Whitney U test were applied to evaluate ICRS II scores and the relative protein expressions. Spearman correlation was used in the comparison of the relationship between PRG4, COL II expression and the tribological parameters. The orthogonal 2 partial least squares (O2PLS) 33-35 regression mathematical model was used in the multivariate analysis, and the Variable Importance in the Projection (VIP) scores of each variable were calculated according to the existed methods 36-38. All data were presented as mean ± SD, and *P*<0.05 was selected as the cut off for statistical significance.

## Results

### Repair of articular cartilage in sheep

**Figure 1:**
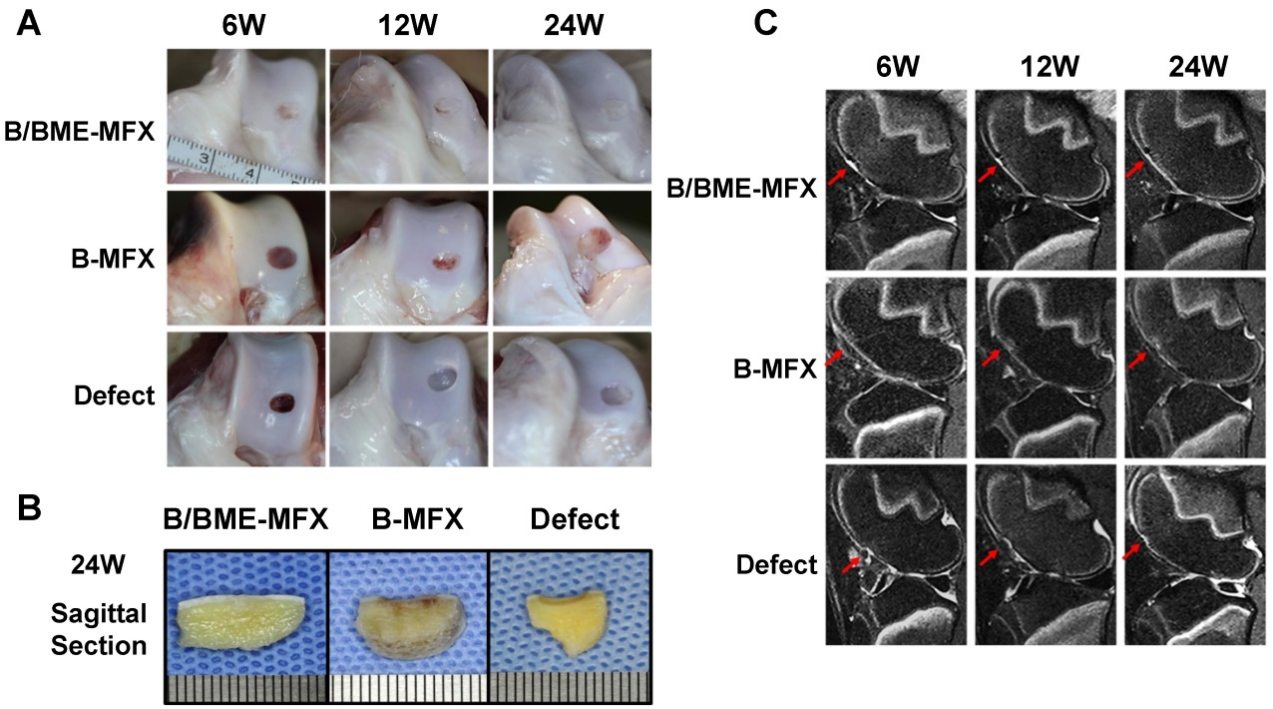
** Gross observation and radiographic assessments on the cartilage repairs between the different groups. (A)** Representative gross observation photographs of the regenerative cartilage after the B/BME-MFX, B-MFX or Defect treatments at the 6th, 12th and 24th weeks. **(B)** Median sagittal section observation of cartilage defect area at the 24th week among the three groups. **(C)** Representative T2 fat-suppressed MRI images of the regenerative cartilage surface (red arrow) at the 6th, 12th and 24th weeks in the B/BME-MFX, B-MFX and Defect groups.

**Figure 2:**
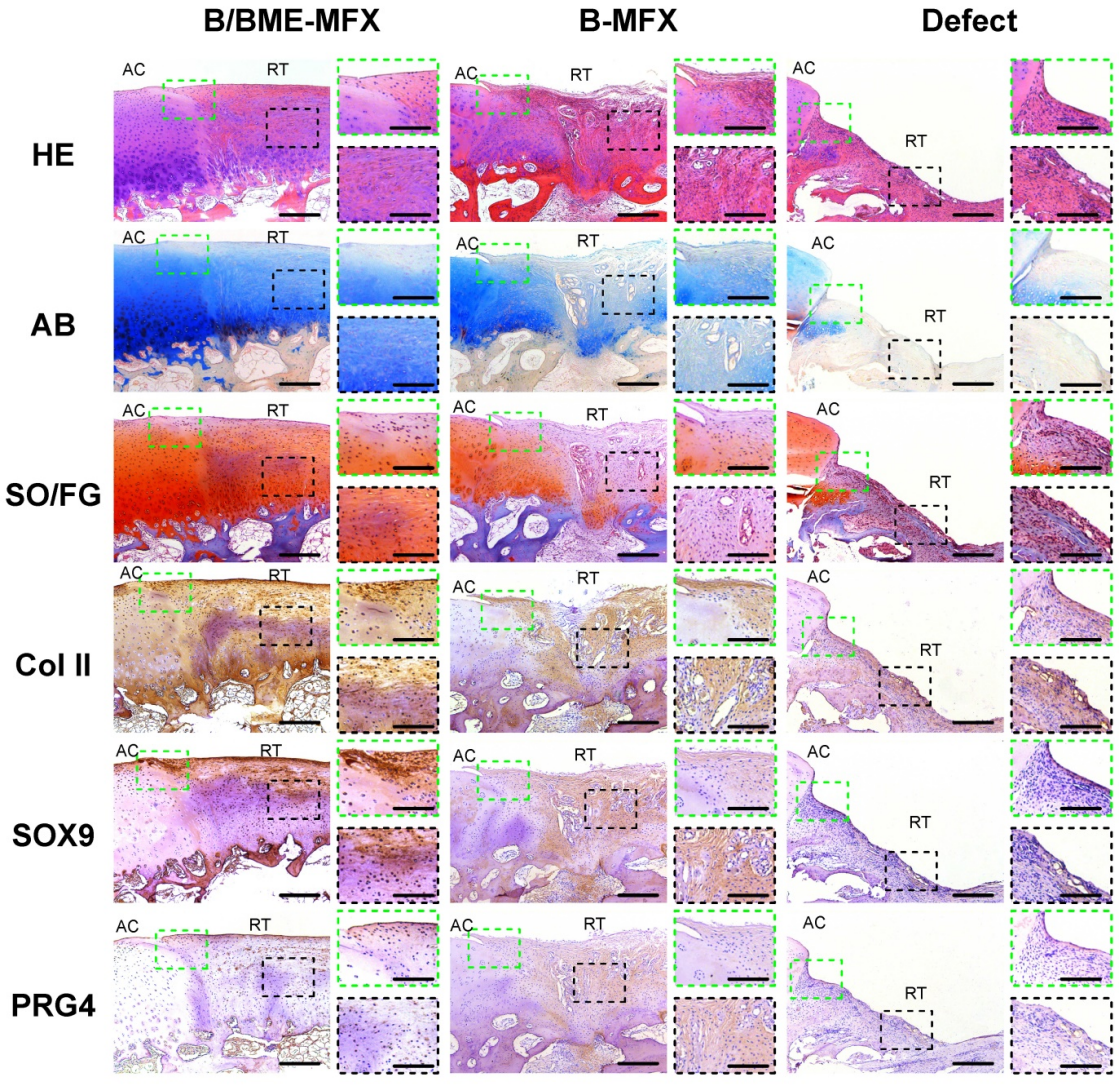
** Histological analyses on the cartilage repairs between the different groups.** Typical histological staining pictures at the end of each treatment, including hematoxylin eosin (HE), AB, SO/FG, as well as IHC stainings against COL II, SOX9 and PRG4 (AC, adjacent cartilage; RT, repair tissue; 100X, scale bar 200 μm); the repair boundary of the cartilage surface (green dashed box) and the middle layer of regenerative tissue (black dashed box) were respectively amplified (400X, scale bar 50 μm).

After 6, 12 and 24 weeks, cartilage repair in the defect region was detected using a series of traditional evaluation methods, including gross observation, radiographic imaging, biomechanical analysis and histological staining. Results showed that a “better-looking” repairing tissue from the B/BME-MFX group was acquired, in which more than 80% of the cartilage defects were filled. A mixture of various proportions of fibrocartilage and hyaline cartilage from B/BME-MFX and B-MFX groups can be found under the microscope (Figures 1-2, S3-6).

### Tribological properties and wear resistance of repaired cartilage

**Figure 3:**
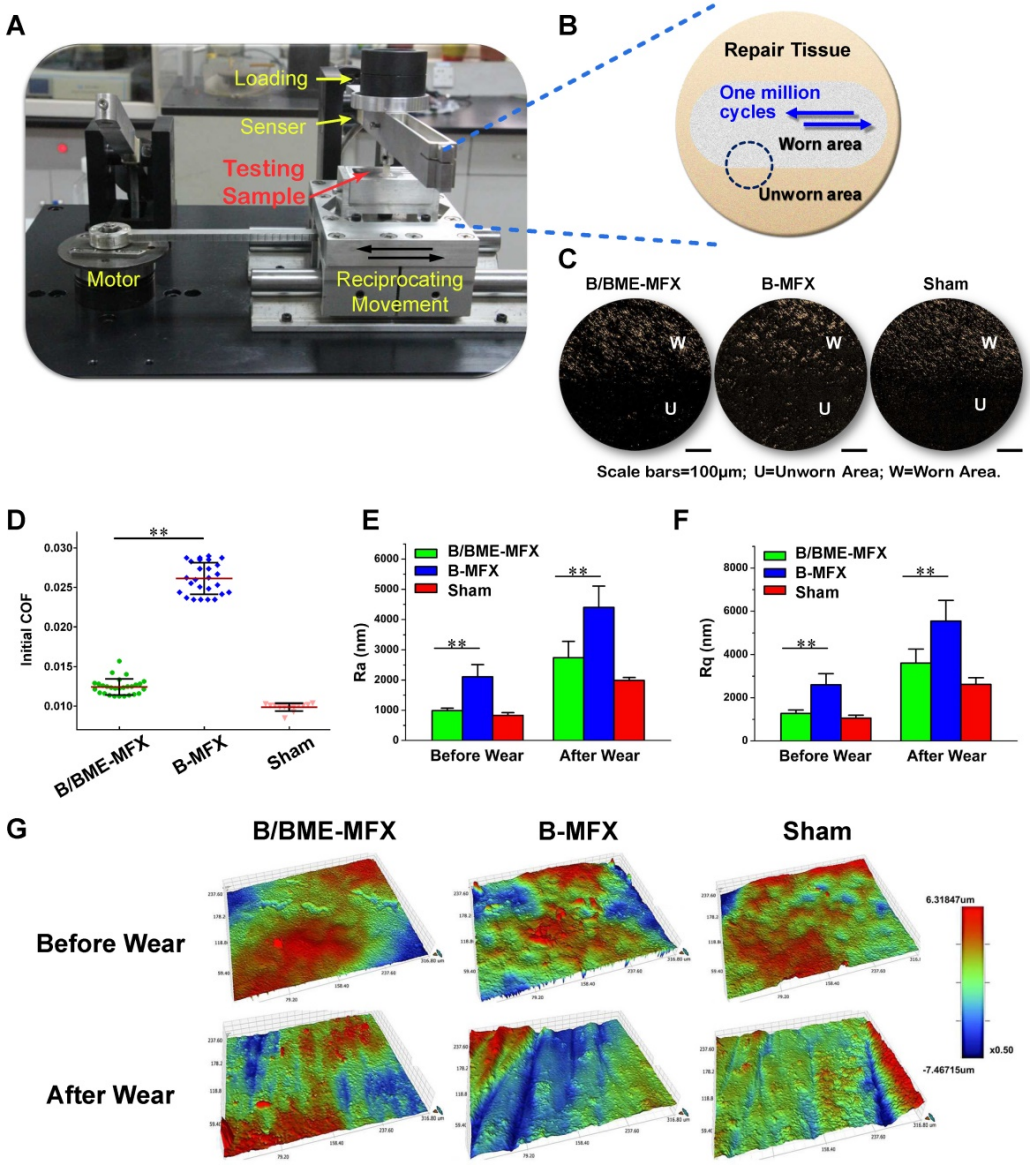
***Ex-vivo* million-cycle reciprocating wear test. (A)** Introduction of the wear test equipment. **(B)** A diagrammatic sketch of the wear test upon the repairing tissue, with the gray section representing the worn area (W), yellow section indicating the unworn area (U), blue arrows showing the reciprocating motion directions, and dotted circle standing for the worn boundary. **(C)** Typical photographs of the worn boundary from each group after the million-cycle wear tests, taken by a dissecting microscope (scale bar 100 μm). **(D)** Comparisons of the initial COF among the B/BME-MFX (n=28), B-MFX (n=26) and Sham (n=12) groups (**P<0.001). **(E-G)** Representative surface topography images of the cartilage repairs before and after the wear test among the three groups, taken by an optical profiler. The surface roughness degree was quantitated by parameters Ra and Rq (**P<0.001, respectively).

A million-cycle reciprocating wear test with real-time COF measurement and subsequent surface morphology assessment was conducted to evaluate the tribological properties of the repaired cartilage. We found that COF of the B/BME-MFX repaired tissue group was very similar to the Sham group, while B-MFX group showed a highest COF of all (Figure 3D, 0.01222±0.0006 *vs* 0.02613±0.0020, *P*<0.001). Distinct boundaries were found between the worn (W) and unworn area (U) under the dissecting microscope (Figure 3C). This roughly indicated a different surface morphology between the two groups, where the W area in the B-MFX repairs displayed the worst surface uniformity. Outlined and quantified by a 3D optical profiler, we found a smoother regenerative surface in the B/BME-MFX group with lower levels of surface roughness compared to the B-MFX samples (Ra=990.4±76.6 *vs* 2116.3±400.2 nm, Rq=1270.6±163.9 *vs* 2601.1±513.7 nm, *P*<0.001, Figures 3E-G). From this point of view, the B/BME-MFX treatment provided an optimal reciprocal surface suitable for the subsequent wear test.

**Figure 4:**
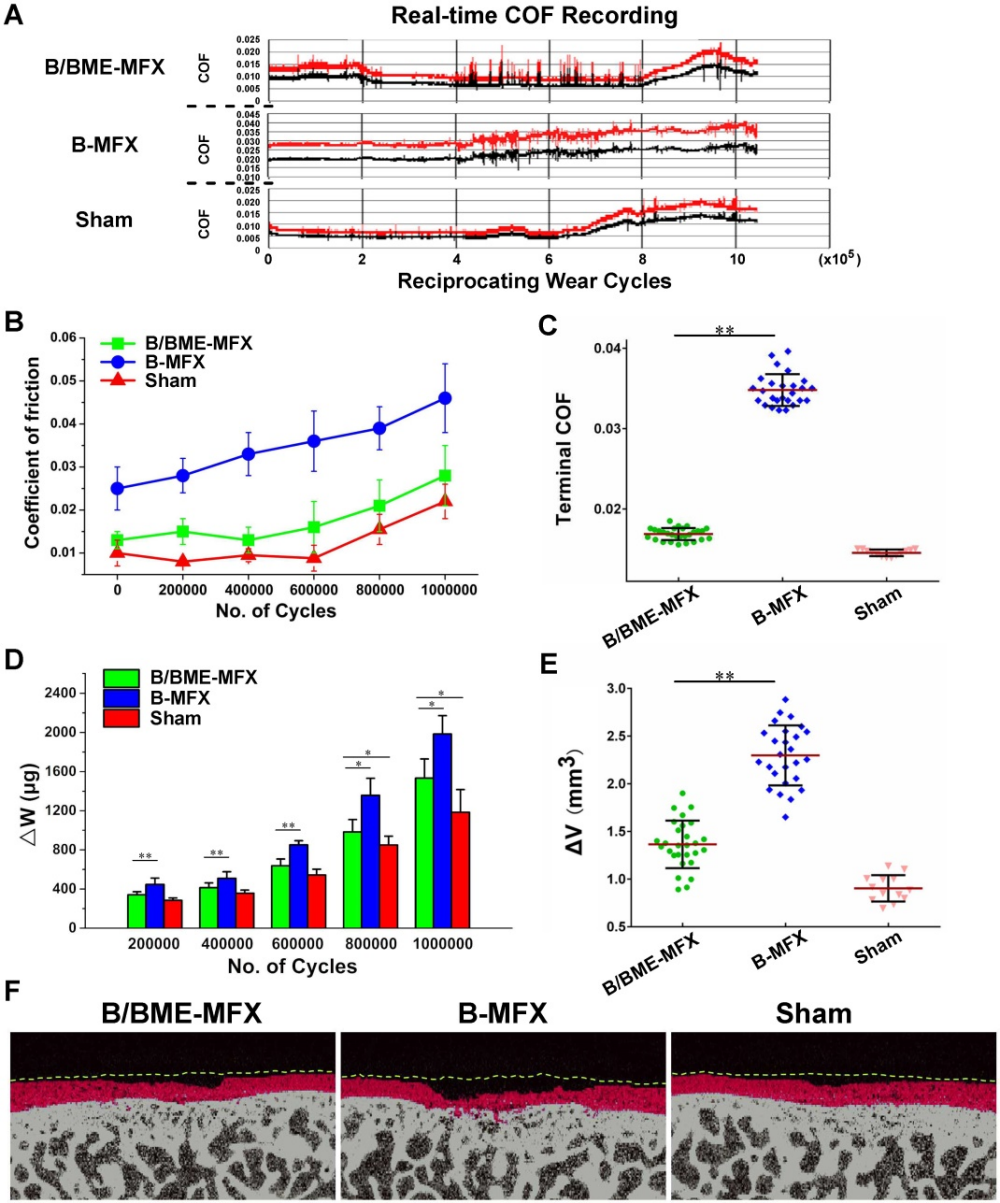
** Impacts on the repairing tissues during and after *ex-vivo* million-cycle wear test. (A)** Real-time COF recording among the B/BME-MFX, B-MFX and Sham groups. COF data were recorded by two electrodes (red and black lines) with 20 times per second and 96 reciprocating wearing cycles per minute. **(B)** Dynamic changing of the mean COF among the three groups were analyzed at every 200,000 cycles. **(C)** Comparisons of the terminal COF among the B/BME-MFX, B/MFX and Sham groups (**P<0.001).** (D)** Analysis of the weight loss (△W) from the grinding cartilage surfaces among the three groups at the corresponding wear cycles (*P<0.05, **P<0.001, respectively). **(E)** Analysis of cartilage volume loss (△V) after the wear test in the three groups (**P<0.001). **(F)** Typical micro-CT images of the repairing tissues from each group, matched according to the location of bone trabecula before and after wear (yellow lines indicated the cartilage border before the wear test, and the red regions stood for the worn area).

On the whole, the COFs of the three groups rose with the increase of cycle number (Figures 4A and B). And the average terminal COF of the B-MFX regenerative tissue was found twice as much as that of the B/BME-MFX group (0.046±0.009 *vs* 0.025±0.007, *P*<0.001, Figure 4C). Likewise, at the end of the wear test, the B-MFX tissues were found to be the most seriously damaged of all, while a relatively flat surface was left in the B/BME-MFX group (Figures 3E-G, Ra=4398.6±708.9* vs* 2742.4±540.2 nm, Rq=5543.2±957.7 *vs* 3604.1±650.9 nm, *P*<0.001).

The wear test process is accompanied with cartilage destruction and tissue loss, which reflects the wear resistant capacity of the repaired tissue. This can be evaluated by the cartilage △W and △V during the whole test. In general, no matter in which time points, △Ws in the B-MFX group were always the highest of all. In the beginning, △Ws of the B/BME-MFX tissues were close to the Sham group. However, at the last two recording points (800,000 and 1,000,000 wear cycles) the gaps between the B/BME-MFX and Sham groups gradually widened (*P*=0.022 and *P*=0.015, respectively, Figure 4D). The cartilage volume loss of each sample was measured using the micro-CT scan before and after wear (Figures 4E and F). When the wear tests ended, the cartilage volume of the B-MFX group altered the most (2.312±0.279 mm^3^), while a low ΔV level of the B/BME-MFX regenerative tissue was comparable in average with the Sham group (1.365±0.249 *vs* 0.904±0.138 mm^3^).

### Correlations between PRG4 expression and tribological properties of repaired cartilage

**Figure 5:**
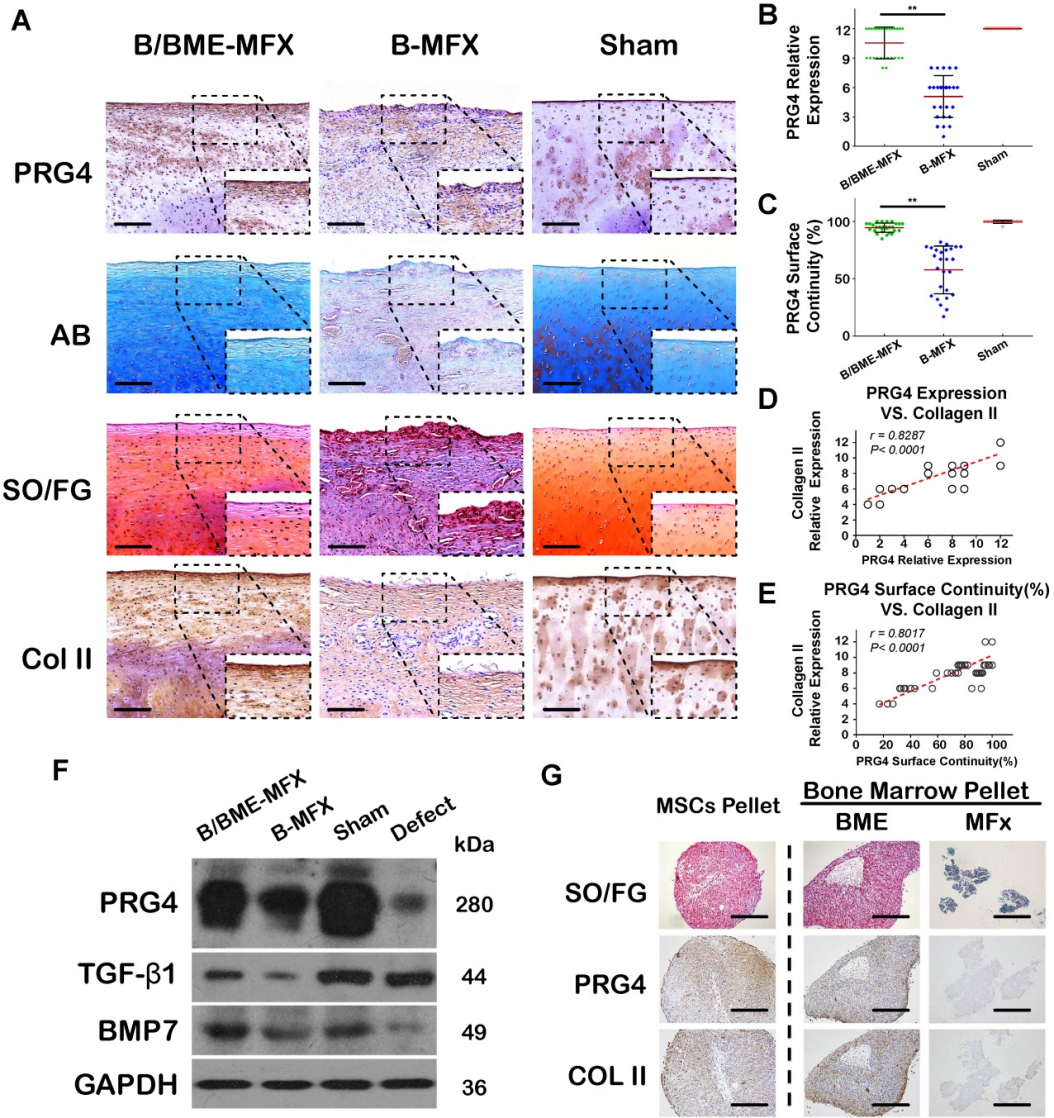
** Component analyses of the regenerative cartilage in the different groups. (A)** Representative images of AB, SO/FG and IHC stainings against PRG4 and COL II among the B/BME-MFX, B-MFX and Sham groups (200X, scale bar 100 μm); the surface layer (black dashed box) of regenerative tissue was amplified (400X, scale bar 50 μm). **(B and C)** Semi-quantitative analyses of PRG4 surface continuity and expression quantity in the different groups (**P<0.001, respectively). **(D and E)** Correlation relationships between COL II relative expression and PRG4 surface continuity (*r* =0.8017, *P*< 0.001) or expression quantity (*r* =0.8287, *P*< 0.001). **(F)** Immunoblotting analyses of PRG4, TGF-β1 and BMP-7 protein expressions in the B/BME-MFX, B-MFX, Sham and Defect groups, the intensity of the target proteins was standardized to GAPDH.** (G)** *In-vitro* bone marrow chondrogenetic pellet culture in the BME and MFX samples after 28 days of TGF-β3-chondrogenetic induction. Typical SO/FG and IHC staining images against PRG4 and COL II in the two groups were compared, parallel induced MSC was determined as the positive control (50X, scale bar 400 μm).

Considering the essential role of PRG4 in joint lubrication, we examined the protein expression of PRG4 in repaired cartilage tissues by IHC and western blot analysis, and test its correlation with the tribological properties of repaired cartilage. We showed that PRG4 was highly and continuously expressed in the superficial zone of the B/BME-MFX regenerative cartilage, but not in the B-MFX tissue surface (Figures 5A-C). Interestingly, both the surface continuity and expression of PRG4 were found to be positively correlated with COL II level of the regenerative cartilage in all the samples (Figures 5D and E).

Bone marrow blood with or without enrichment were separately pellet-cultured for chondrogenesis* in vitro*, which simulates the *in vivo* B/BME-MFX and B-MFX group. The staining findings showed that the ability of the bone marrow after enrichment in generating extracellular matrix with PRG4 protein was superior to the microfracture alone treatment (Figure 5G). There existed a similar trend of the upregulation of PRG4, BMP-7 and TGF-β1 in the B/BME-MFX regenerative tissue compared to the B-MFX group (Figure 5F).

**Figure 6:**
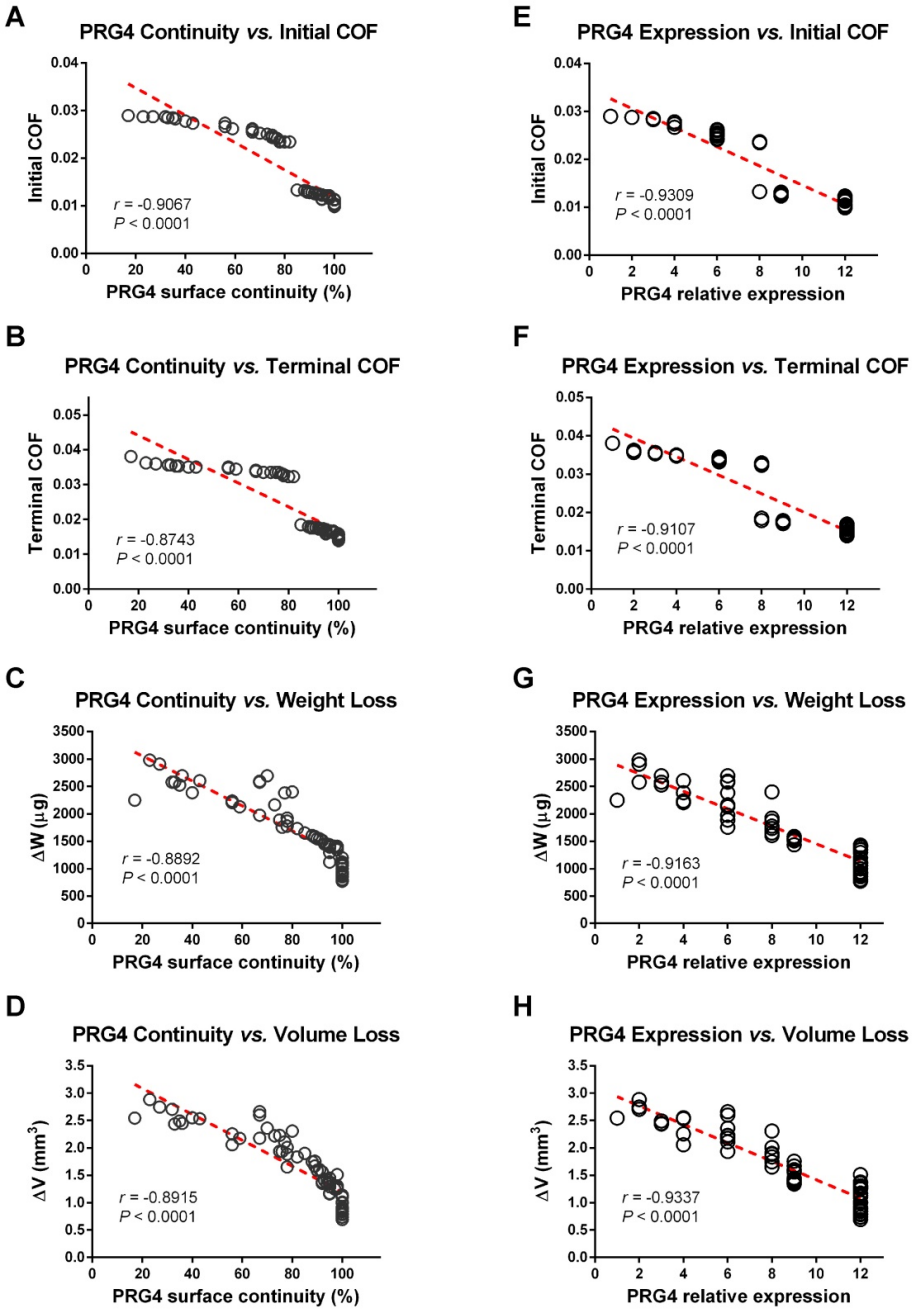
** Correlation relationships between various tribological parameters and PRG4. (A-D)** PRG4 surface continuity was found negatively correlated with initial and terminal COF, △V and △W (*P* < 0.0001, respectively). **(E-H)** PRG4 expression quantity was also found negatively correlated with initial and terminal COF, △V and △W (*P* < 0.0001, respectively).

Next, we separately compared the relationships between PRG4 expression levels and various tribological parameters in all samples (Figures 6 and S7). The initial COF and roughness factors (Ra and Rq) were found negatively correlated both with PRG4 surface continuity and expression quantity, which means a good PRG4 expression level represents a low-friction regenerative cartilage surface ready for wear. Furthermore, negative correlations were also definitely detected between PRG4 and the terminal COF, △V, △W as well as after wear Ra and Rq.

### Relationships between PRG4 expression and wear resistant properties of repaired cartilage

**Figure 7:**
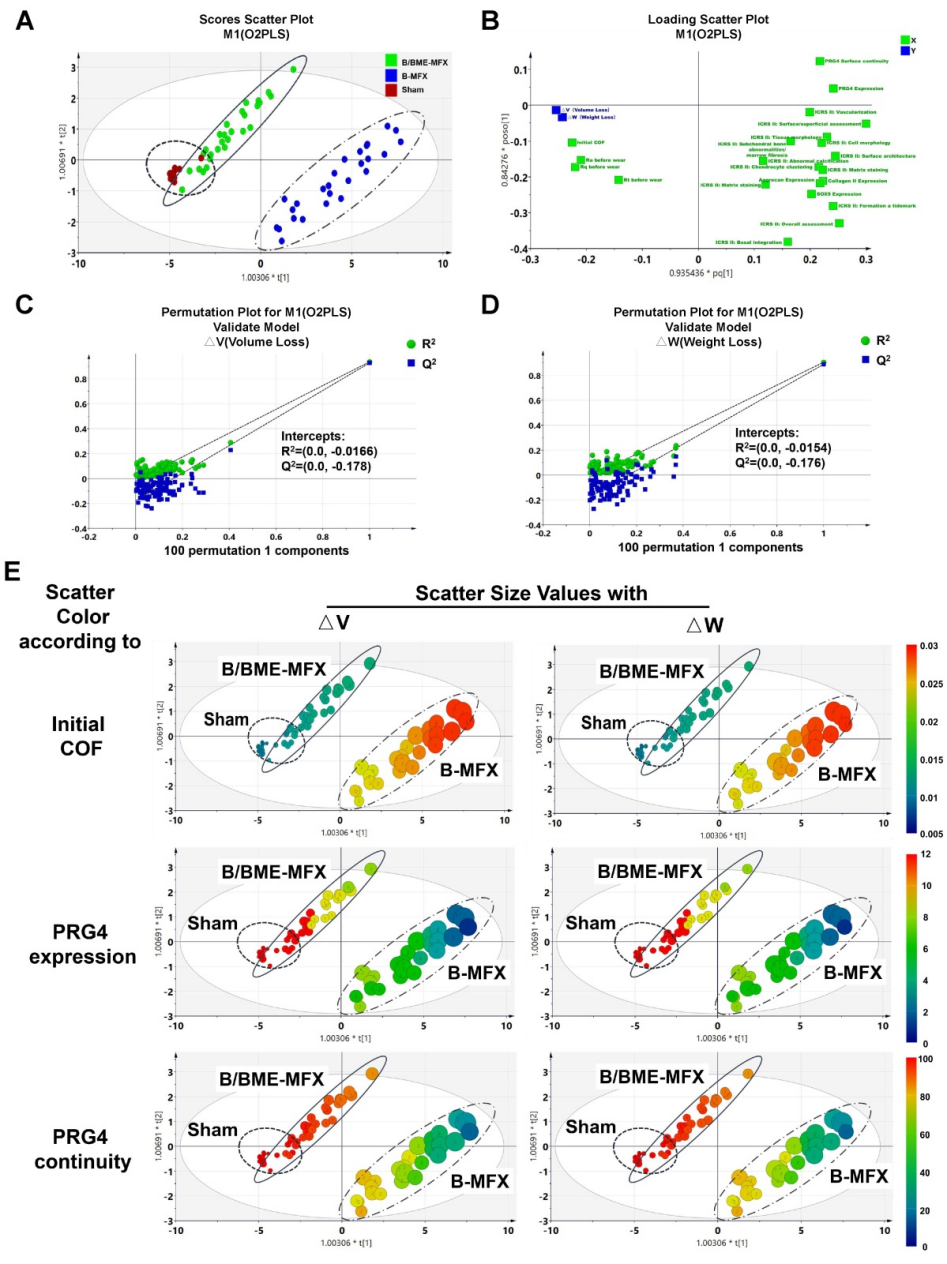
** O2PLS multivariate analyses on wear resistance evaluation of the regenerative cartilage. (A)** O2PLS scatter plot showed the distribution of all the independent variables among the B/BME-MFX (full line circle), B-MFX (dotted line circle) and Sham (dashed line circle) groups, △V and △W were defined as two dependent variables. **(B)** O2PLS loadings plot illustrated the covariance relationships between all the variables. **(C and D)** Validity verifications of the original model. **(E)** Scatter plots displayed the relationships and distributions between △V/△W and the independent variables among the three groups, with the dot size referring to the volume/weight loss amount, and blue to red indicating the increase of variable quantitative value.

Subsequently, a multivariate statistical model was established to explore whether PRG4 can act as a straightforward biomarker to predict the wear resistant property of repaired cartilage. Considering that there may exist multicollinearity among variables, we employed the VIP scores under the partial least squares (PLS) regression rather than the stepwise regression methods. We collected all of the quantifiable parameters relevant to cartilage regeneration evaluation as independent variables (X_n_), including the quantity and surface continuity of PRG4 expression, the relative expression of COL II, SOX-9 and aggrecan,13 items of ICRS II scoring and tribological parameters such as initial COF and surface roughness factors before wear. We identified △V and △W as two dependent variables (Y_1_ and Y_2_) reflecting the wear resistant property and set up an O2PLS regression mathematical model. Scores scatter plot showed that this model performed well in distinguishing between the B/BME-MFX and B-MFX samples on wear resistant performance (Figure 7A).

A supplementary loading plot was presented to show the overall distribution of the included variables, and similar correlation tendencies as the linear regression can be found in the O2PLS model (Figure 7B). We also used permutation plots as a quality control that strongly indicated the validity of the original model (Figures 7C and D). The scatter plots illustrated a large overlap between the B/BME-MFX and Sham groups in most of the independent variables within the low △V/△W ranges, while the B-MFX samples showed a large difference from them (Figures 7E, Figures S8-11).

**Figure 8:**
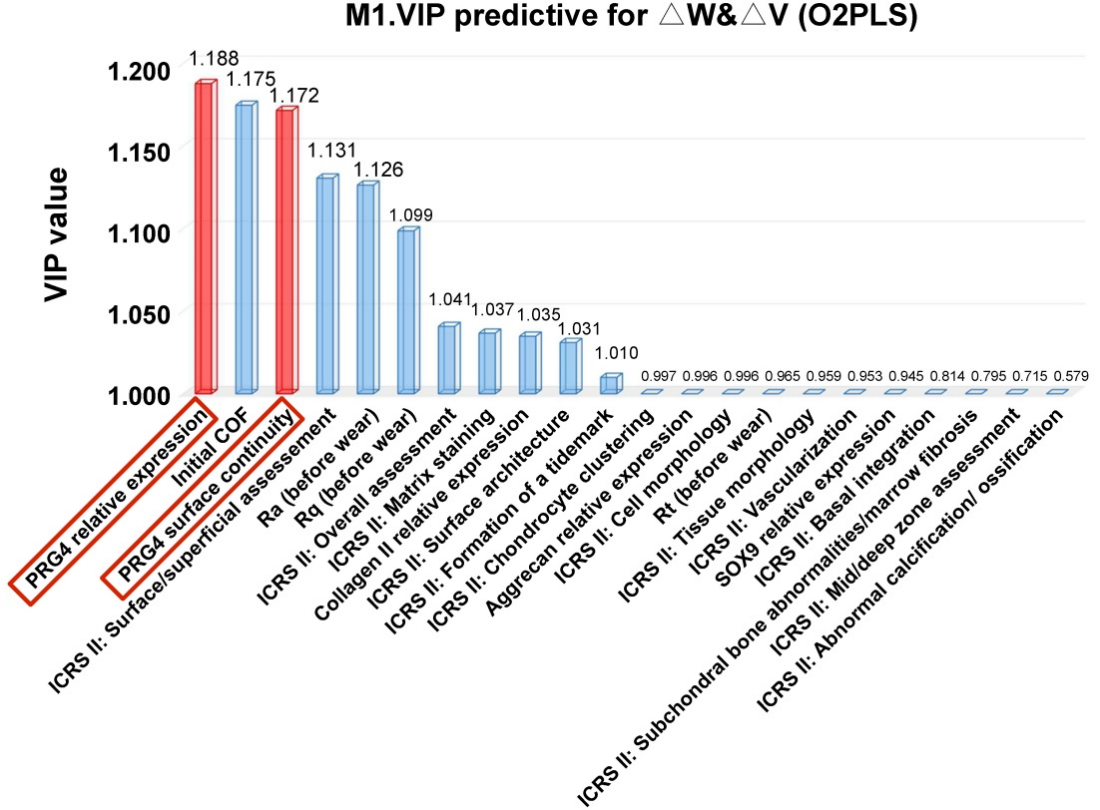
** Variable Importance for the Projection (VIP) predicting analysis.** Data presented the ranking of all the relevant ICRS II and tribological parameters according to the VIP predictive analysis for dependent variables △V and △W in the O2PLS model; VIP values greater than 1.0 indicated “important” variables; two PRG4 relevant factors were highlighted red.

As a useful and simple strategy tool for the importance evaluation of each variable in the above O2PLS model, the VIP score manifested that among numerous parameters, PRG4 expression quantity and surface continuity ranked at the top, suggesting that these two factors will effectively predict the deformation and durability of the regenerative cartilage under the friction load (Figure 8).

## Discussion

The surface of healthy articular cartilage is endowed with smooth and durable properties, where the surface coefficient of friction is almost zero (as low as 0.0005) 39, 40. Movement between the articular surface is an integration of multi-axes motions that includes rolling, sliding, lateral movement and rotation 41. Under the complicated circumstances of constantly changing kinematic velocity and bearing pressure, this surface can help joints to function for decades 42. In this study, we have shown for the first time the comprehensive profiles of tribological properties of repaired cartilage that can determine the long-term durability of weight bearing. We showed that PRG4 is an ideal biomarker for the prediction of tribological and wear resistant properties of repaired cartilage tissue.

Histological assessment has been a fundamental criterion evaluation of cartilage repair. ICRS I and ICRS II scoring systems has been used as bench marks in the clinical practice. However, these criteria are not able to provide information on wear resistance of the repairing tissue, which directly translates to the durability of the regenerative cartilage. To mimic the *in vivo* locomotion pattern between the articular interface, we have established a program of an *ex vivo* million-cycle reciprocating wear. This will enable examination of a series of tribological parameters for repaired tissues from different cartilage repair strategies. Our results have shown that after the wear test, the B/BME-MFX repaired cartilage outperformed the B-MFX competitor in terms of representative tribological parameters including surface COF, roughness and wear resistance indicators such as △V and △W. However, these experiments are so lengthy and complicated that not every clinician or scientist could afford the time or equipment to accomplish them. Therefore, a preferable biomarker that can be easily tested and able to accurately represent the wear resistant property is urgently required.

The long-term integrity of an articulation depends on the nourishment of its cartilage component and the protection of the cartilage surface from the inevitable mechanical abrasion. It has been recognized that PRG4, hyaluronic acid (HA), and surface active phospholipids (SAPL) are three major constituents in the synovial joint providing the articular cartilage with low-friction and low-wear properties 43-45. PRG4, also known as superficial zone protein (SZP) and lubricin 46, can be synthesized by the flat chondrocytes in the superficial zone of the articulating cartilage. This polymer, containing an extensive mucin-like region substituted by O-linked oligosaccharides, can reduce friction through the development of repulsive forces 47. Joint friction is elevated and accompanied by accelerated cartilage damage in humans and mice that have genetic deficiency of lubricin 19, 48. Chan *et al.* 49 have demonstrated that PRG4 plays an intrinsic and critical role in the cartilage boundary lubrication, whereas the effects of HA and SAPL on the tribological behavior are marginal. Besides, the study of Majd *et al.* 50 has reported that HA helps to keep PRG4 at the cartilage surface, but this interaction is unstable and can be interfered by albumin in the synovial fluid; By contrast, collagen II fibrils maintain PRG4 at the cartilage-cartilage interface *in situ*. Similar findings were discovered in our study that between PRG4 and COL II there existed a synergistic protein expression pattern and a positive correlation relationship.

It has been reported in the previous studies that morphogens like BMP-7, or growth factors especially TGF-β1, can effectively increase the expression and accumulation of PRG4 in the chondrocytes 51, 52. Our findings have revealed a similar upregulated trend of expression between PRG4 and these upstream proteins both in the B/BME-MFX regenerative tissue as well as the normal cartilage. This may attribute to the preparation of the beneficial microenvironment from the bone marrow enrichment technique towards PRG4 overexpression in the B/BME-MFX samples.

**Figure 9:**
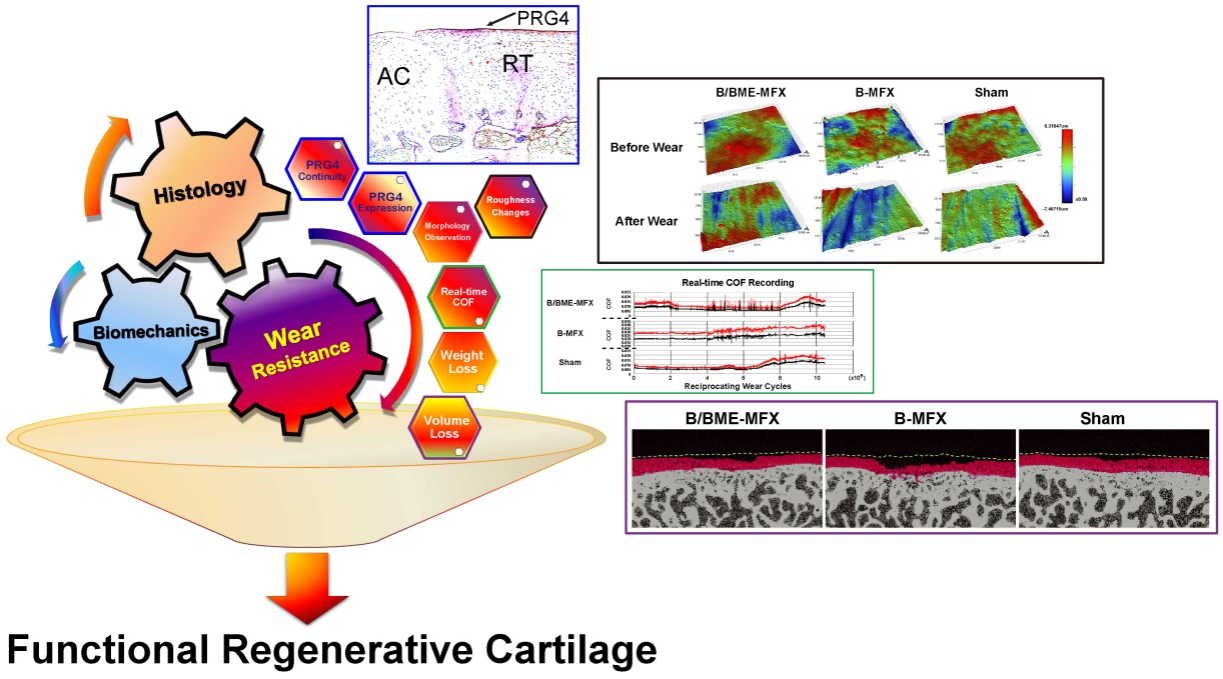
** A novel comprehensive evaluation system of the regenerative cartilage.** A systemic evaluation of the functional regenerative cartilage obtained from a successful cartilage repair should cover traditional histological assessment, conventional biomechanical testing, as well as the wear resistance evaluation especially PRG4 expression quantity and surface continuity.

In this study, we demonstrated that PRG4 expression can predict the terminal wear resistant performance of the regenerative cartilage, especially in the interpretation of the weight and volume loss, suggesting what kind of regenerative tissue can finally survive in the harsh wear test. Underlying mechanisms of PRG4 in wear resistance may attribute to its prevention of chondrocyte apoptosis 53 and modulating the viscoelastic properties with HA in synovial fluid 54. Detection of PRG4 enriches the functional cartilage regeneration evaluation system previously based on ICRS II scoring (Figure 9).

In conclusion, we have successfully established a comprehensive wear resistance evaluation system and revealed that PRG4 expression was a useful and convenient predictor of good cartilage repair. Arthroscopic biopsy and intraoperative frozen detection of PRG4 are accessible in today's orthopedic surgery. In the future, more non-invasive approaches in detecting PRG4 will be further investigated and validated, such as the targeting PRG4 probe for PET/MR imaging. The promising integration of the traditional evaluation criteria with PRG4 is expected to guide clinical practice for cartilage repair and benefit more patients in the future.

## Supplementary Material

Supplementary figures 1-6.

Supplementary figures 7-12.
