# Supplementary material for: Proteoglycan 4 predicts tribological properties of repaired cartilage tissue
Source: Theranostics. 2020 Jan 22;10(6):2538–52. doi: 10.7150/thno.39386 (PMC7052906; doi:10.7150/thno.39386)
Supplement: Supplementary file 1 — Supplementary figures 1-6. [file thnov10p2538s1.pdf]

Supplementary Files 1

Supplementary Figures and Legends

Figure S1. Flow chart of tribological evaluations for regenerative cartilage.

Figure S2. Study design and different treatments of B/BME-MFX, B-MFX and Defect groups. (A and B) Surgery design and cartilage repair schematic diagram in the different groups: blue square with white stripes showed the Bio-Gide scaffold, black arrows indicated the exudation direction of the bone marrow blood after the microfracture, red globules stood for the MSCs in the bone marrow blood from the microfracture, yellow triangles represented the MSCs obtained from the bone marrow enrichment technique.

Figure S3. ICRS II scoring evaluation of the regenerative cartilage in B/BME-MFX (n=28), B-MFX (n=26), Defect (n=12), Sham (n=12) groups after a 24-week treatment, including items of overall assessment **(A)**, surface/superficial assessment **(B)**, and surface architecture **(C)** (*P<0.05, respectively).

Figure S4. ICRS II scoring evaluation of the regenerative cartilage in B/BME-MFX (n=28), B-MFX (n=26), Defect (n=12), Sham (n=12) groups after a 24-week treatment, including items of Tissue morphology **(A)**, Matrix staining **(B)**, Cell morphology **(C)**, Chondrocyte clustering **(D)**, Formation of a tidemark **(E)**, and Basal

integration **(F)** (**P*< 0.05, respectively).

Figure S5. ICRS II scoring evaluation of the regenerative cartilage in B/BME-MFX (n=28), B-MFX (n=26), Defect (n=12), Sham (n=12) groups after a 24-week treatment, including items of Subchondral bone abnormalities/marrow fibrosis **(A)**, Inflammation **(B)**, Abnormal calcification/ossification **(C)**, Vascularization **(D)**, and Mid/deep zone assessment **(E)** (**P*< 0.05, respectively).

Figure S6. Biomechanical assessments of the regenerative cartilage. (A) Representative images of the surface elasticity measurement among the B/BME-MFX, B-MFX and Sham groups by using AFM and Young's modulus topography. Both the surface Young's modulus **(B)** and overall compression modulus **(C)** of the B/BME-MFX repairs were closer to the Sham group, and higher than the B-MFX group (**P* < 0.05).

Figure S1

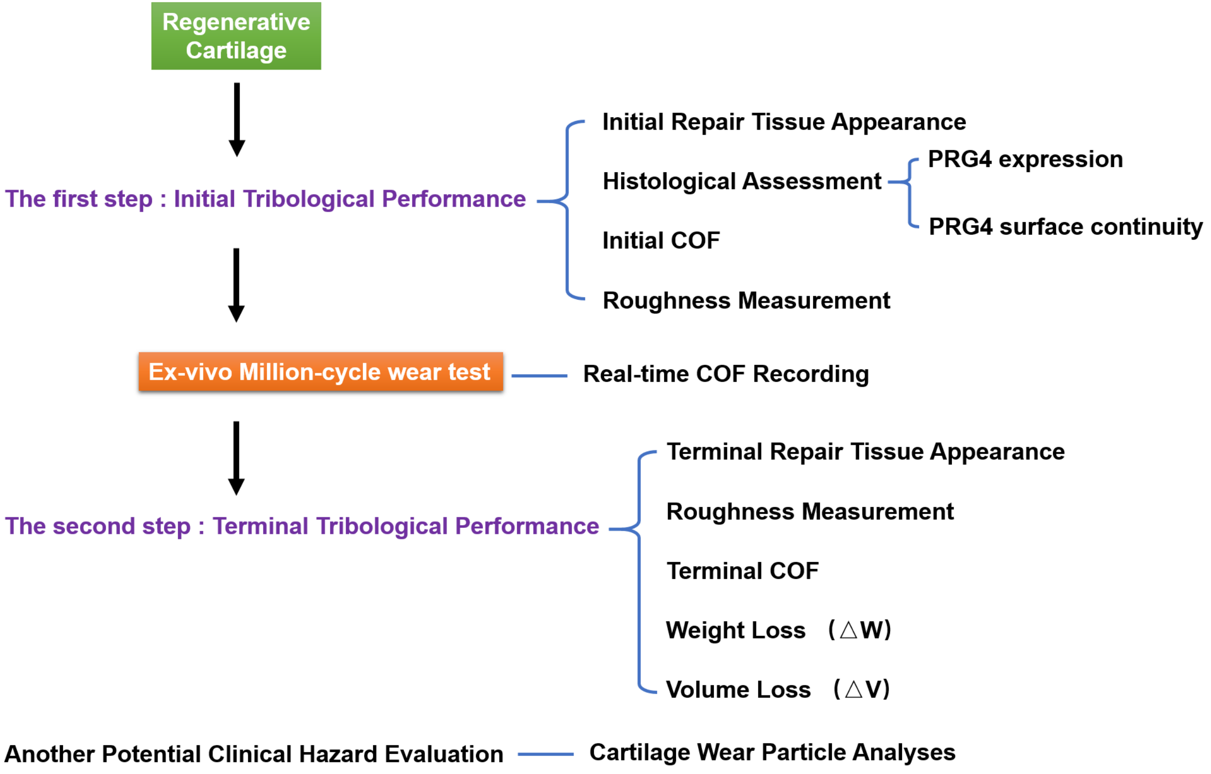

Regenerative Cartilage
The first step : Initial Tribological Performance
Initial Repair Tissue Appearance
Histological Assessment
PRG4 expression
PRG4 surface continuity
Initial COF
Roughness Measurement
Ex-vivo Million-cycle wear test
Real-time COF Recording
The second step : Terminal Tribological Performance
Terminal Repair Tissue Appearance
Roughness Measurement
Terminal COF
Weight Loss (△W)
Volume Loss (△V)
Another Potential Clinical Hazard Evaluation
Cartilage Wear Particle Analyses

Figure S2

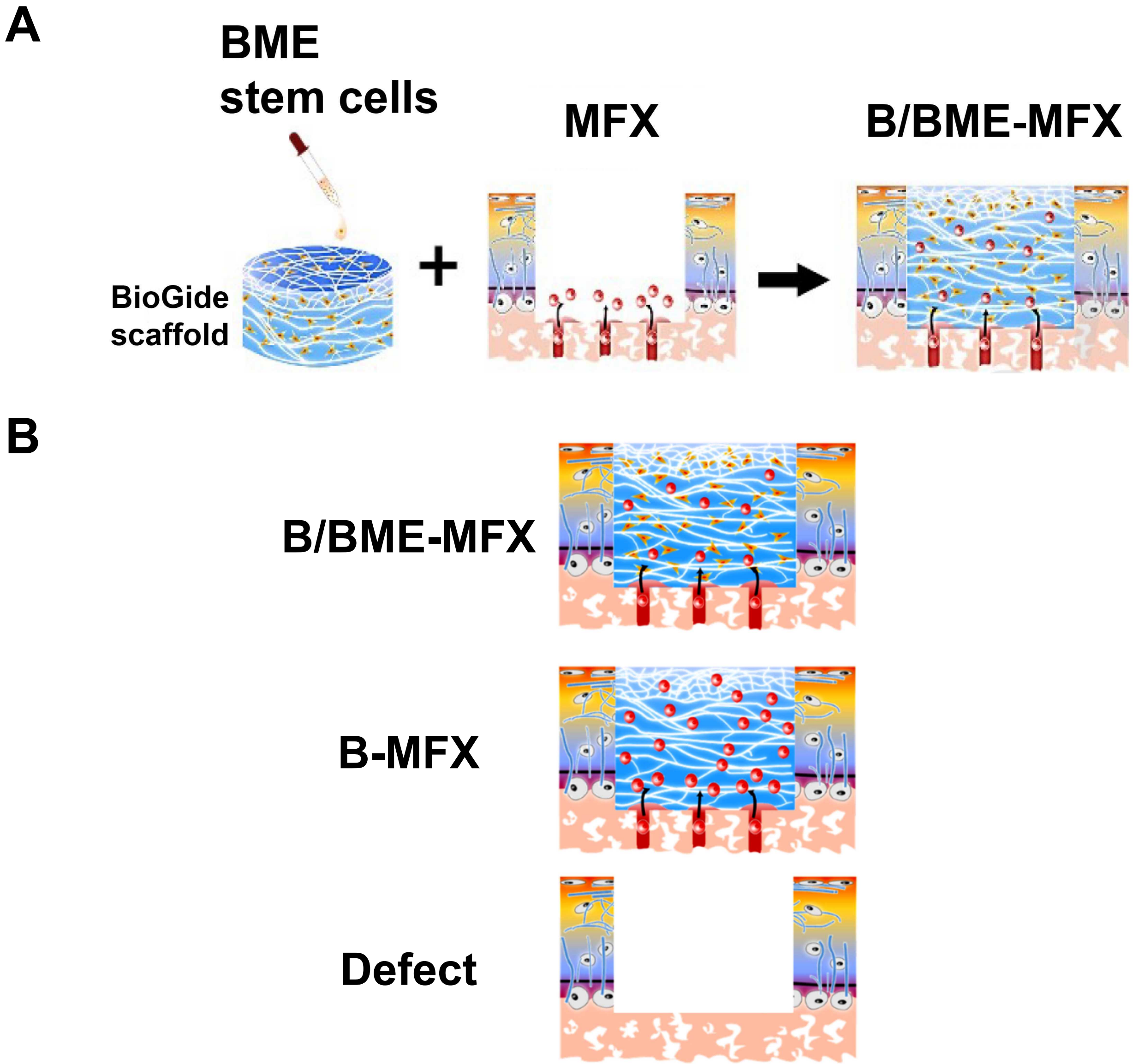
A
BME
stem cells
MFX
B/BME-MFX
BioGide
scaffold
B
B/BME-MFX
B-MFX
Defect

Figure S3

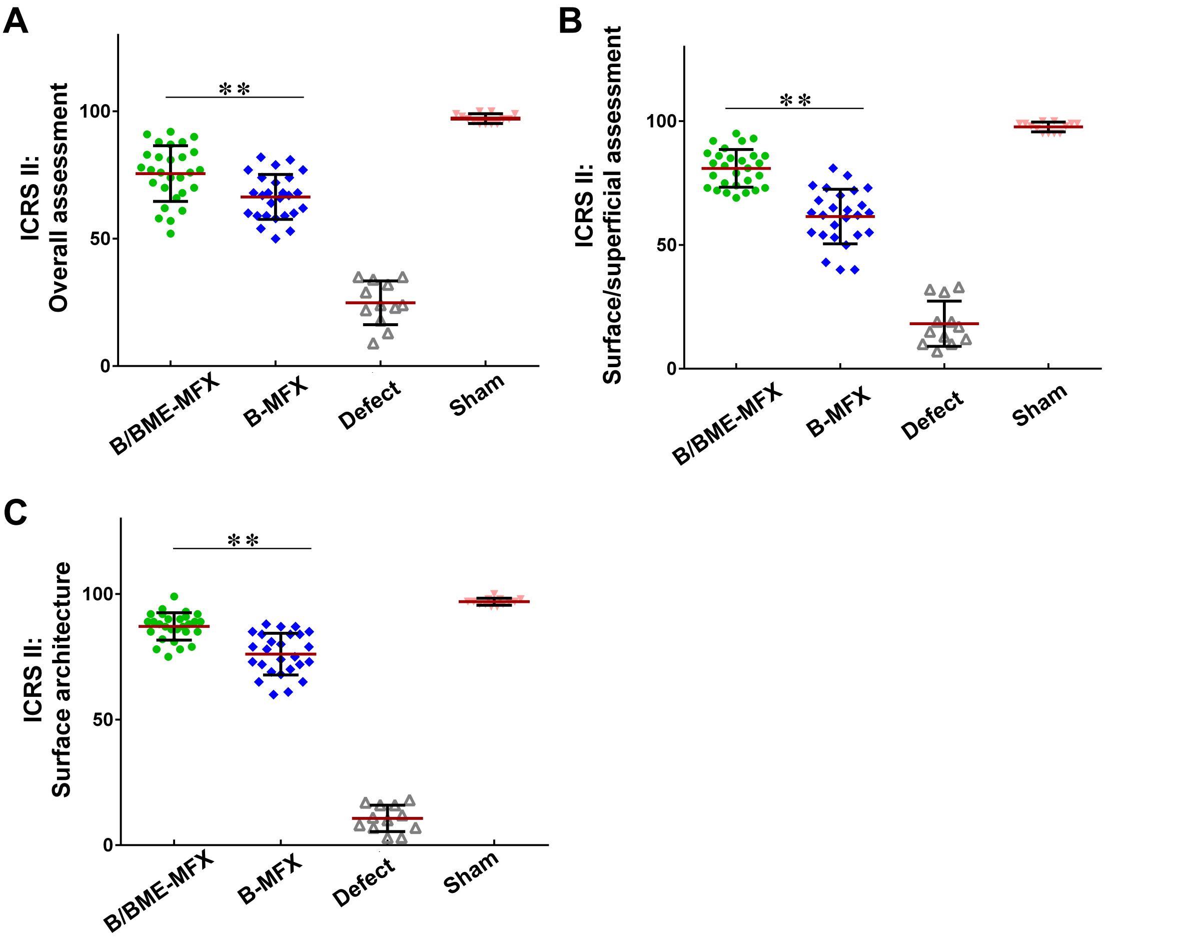
A
ICRS II:
Overall assessment
100
50
0
**
B/BME-MFX
B-MFX
Defect
Sham
B
ICRS II:
Surface/superficial assessment
100
50
0
**
B/BME-MFX
B-MFX
Defect
Sham
C
ICRS II:
Surface architecture
100
50
0
**
B/BME-MFX
B-MFX
Defect
Sham

Figure S4

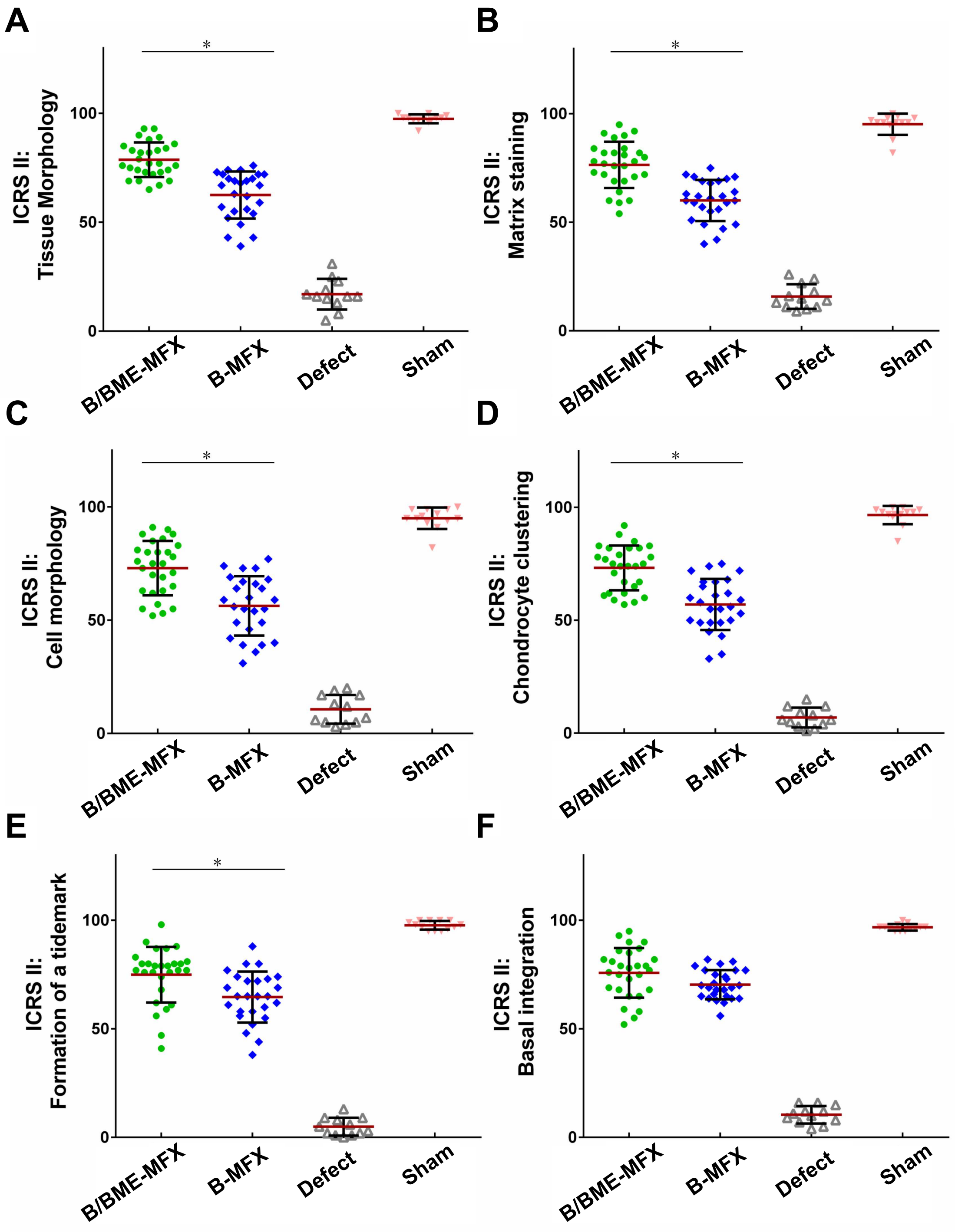

A
*
ICRS II: Tissue Morphology
0
50
100
B/BME-MFX
B-MFX
Defect
Sham
B
*
ICRS II: Matrix staining
0
50
100
B/BME-MFX
B-MFX
Defect
Sham
C
*
ICRS II: Cell morphology
0
50
100
B/BME-MFX
B-MFX
Defect
Sham
D
*
ICRS II: Chondrocyte clustering
0
50
100
B/BME-MFX
B-MFX
Defect
Sham
E
*
ICRS II: Formation of a tidemark
0
50
100
B/BME-MFX
B-MFX
Defect
Sham
F
ICRS II: Basal integration
0
50
100
B/BME-MFX
B-MFX
Defect
Sham

Figure S5

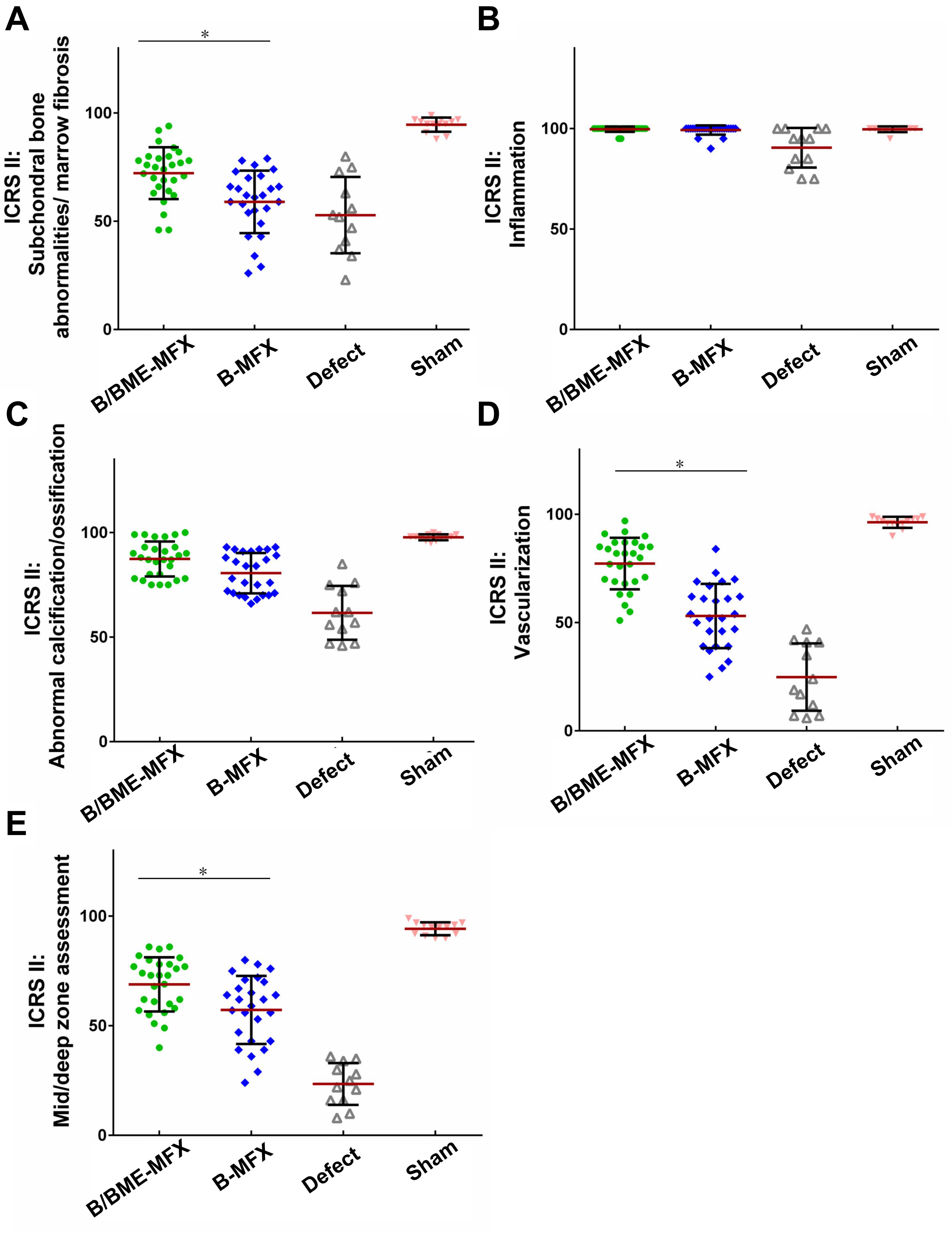

A
B
C
D
E
ICRS II: Subchondral bone abnormalities/ marrow fibrosis
ICRS II: Inflammation
ICRS II: Abnormal calcification/ossification
ICRS II: Vascularization
ICRS II: Mid/deep zone assessment
0
50
100
*
B/BME-MFX
B-MFX
Defect
Sham

Figure S6

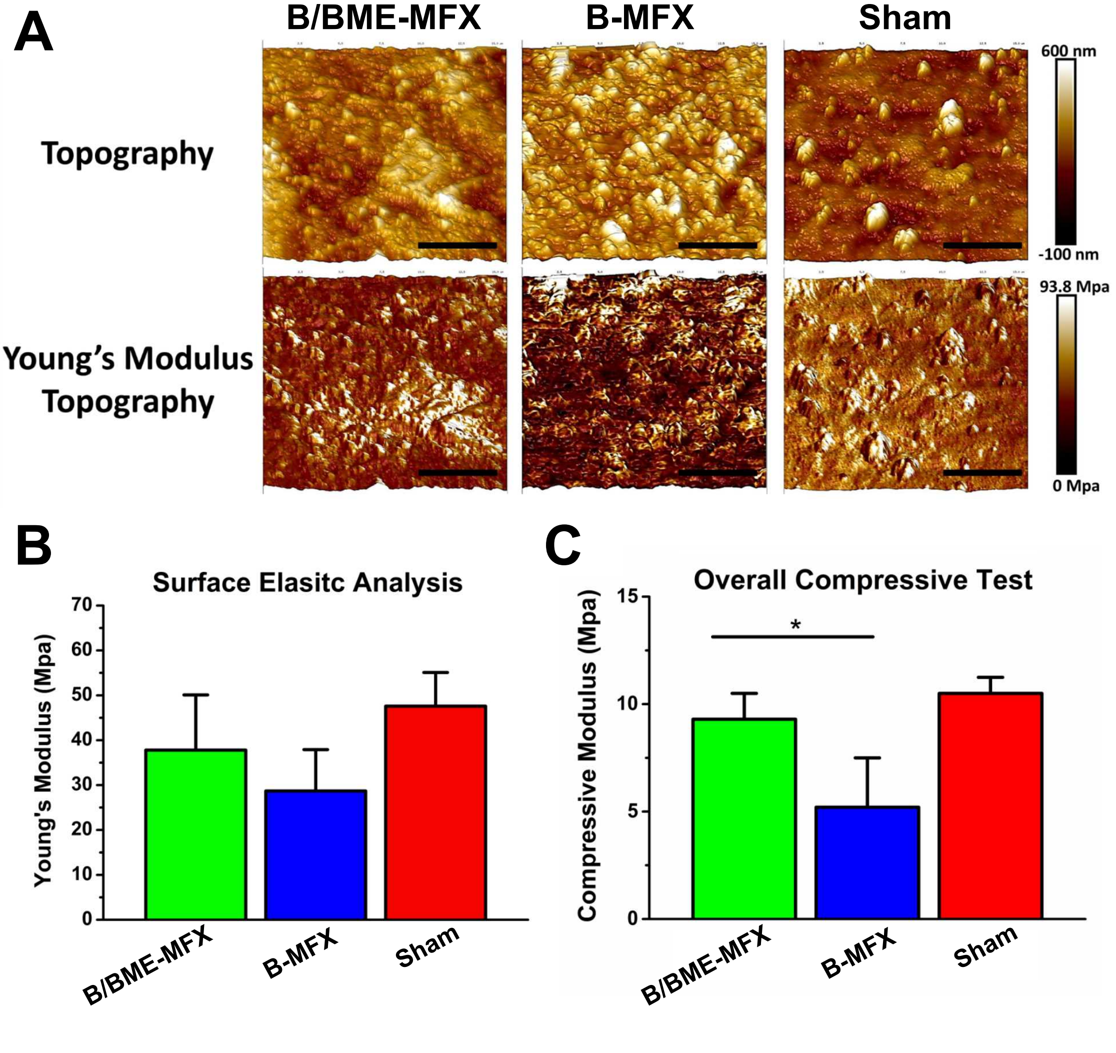

A
B/BME-MFX
B-MFX
Sham
Topography
Young's Modulus Topography
600 nm
-100 nm
93.8 Mpa
0 Mpa
B
Surface Elasitc Analysis
Young's Modulus (Mpa)
0
10
20
30
40
50
60
70
B/BME-MFX
B-MFX
Sham
C
Overall Compressive Test
Compressive Modulus (Mpa)
0
5
10
15
*
B/BME-MFX
B-MFX
Sham
