# Supplementary material for: Proteoglycan 4 predicts tribological properties of repaired cartilage tissue
Source: Theranostics. 2020 Jan 22;10(6):2538–52. doi: 10.7150/thno.39386 (PMC7052906; doi:10.7150/thno.39386)

Supplementary Files 2

Supplementary Figures and Legends

Figure S7. Correlation relationships between PRG4 and surface roughness parameters. (A-D) PRG4 surface continuity was found negatively correlated with Ra and Rq before and after wear ($P < 0.0001$, respectively). **(E-H)** PRG4 expression quantity was also found negatively correlated with Ra and Rq before and after wear ($P < 0.0001$, respectively).

Figure S8-S11. O2PLS multivariate model scatter plots displayed the relationship and distribution between △V/△W and independent variables in the three groups, with the dot size referring to the volume/weight loss amount, and blue to red indicating the increase of variable quantitative value.

Figure S12. IHC images of negative control stainings of PRG4 and SOX9 antibodies in normal cartilage (400X, scale bar 50 μm). Rabbit polyclonal antibody PRG4 and only rabbit serum was respectively used as primary antibody (upper row). Mouse monoclonal antibody SOX9 and only mouse serum was respectively used as primary antibody (bottom row).

Figure S7

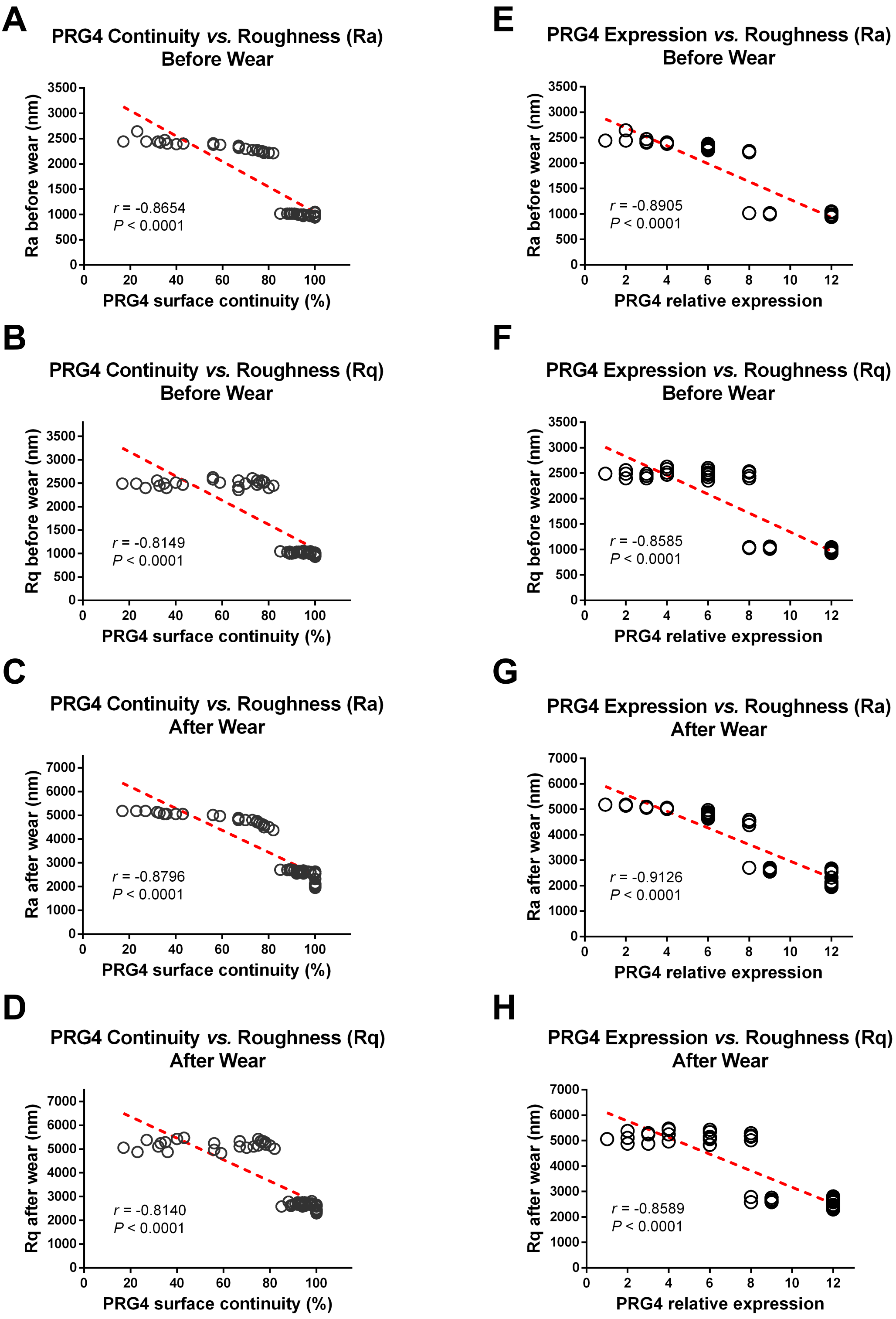
A
PRG4 Continuity vs. Roughness (Ra)
Before Wear
Ra before wear (nm)
PRG4 surface continuity (%)
r = -0.8654
P < 0.0001
E
PRG4 Expression vs. Roughness (Ra)
Before Wear
Ra before wear (nm)
PRG4 relative expression
r = -0.8905
P < 0.0001
B
PRG4 Continuity vs. Roughness (Rq)
Before Wear
Rq before wear (nm)
PRG4 surface continuity (%)
r = -0.8149
P < 0.0001
F
PRG4 Expression vs. Roughness (Rq)
Before Wear
Rq before wear (nm)
PRG4 relative expression
r = -0.8585
P < 0.0001
C
PRG4 Continuity vs. Roughness (Ra)
After Wear
Ra after wear (nm)
PRG4 surface continuity (%)
r = -0.8796
P < 0.0001
G
PRG4 Expression vs. Roughness (Ra)
After Wear
Ra after wear (nm)
PRG4 relative expression
r = -0.9126
P < 0.0001
D
PRG4 Continuity vs. Roughness (Rq)
After Wear
Rq after wear (nm)
PRG4 surface continuity (%)
r = -0.8140
P < 0.0001
H
PRG4 Expression vs. Roughness (Rq)
After Wear
Rq after wear (nm)
PRG4 relative expression
r = -0.8589
P < 0.0001

Figure S8

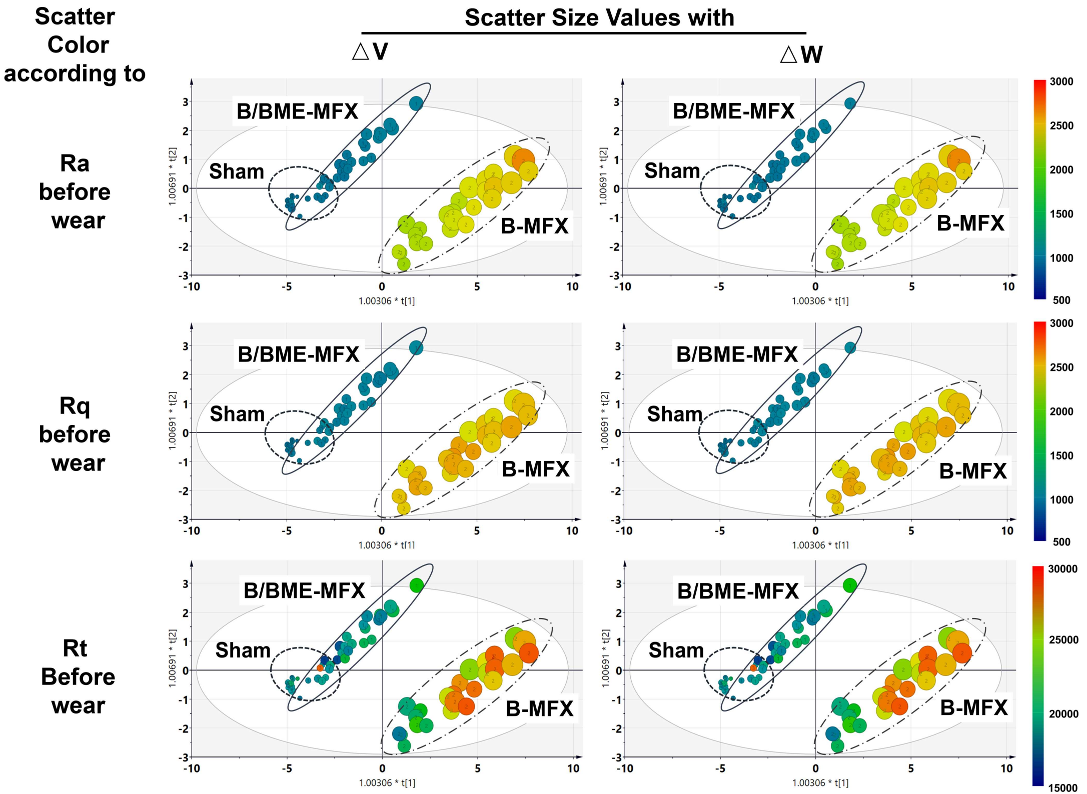

Scatter Color according to
Scatter Size Values with
△V
△W
Ra before wear
Rq before wear
Rt Before wear
B/BME-MFX
Sham
B-MFX
1.00306 * t[1]
1.00691 * t[2]
3000
2500
2000
1500
1000
500
30000
25000
20000
15000

Figure S9

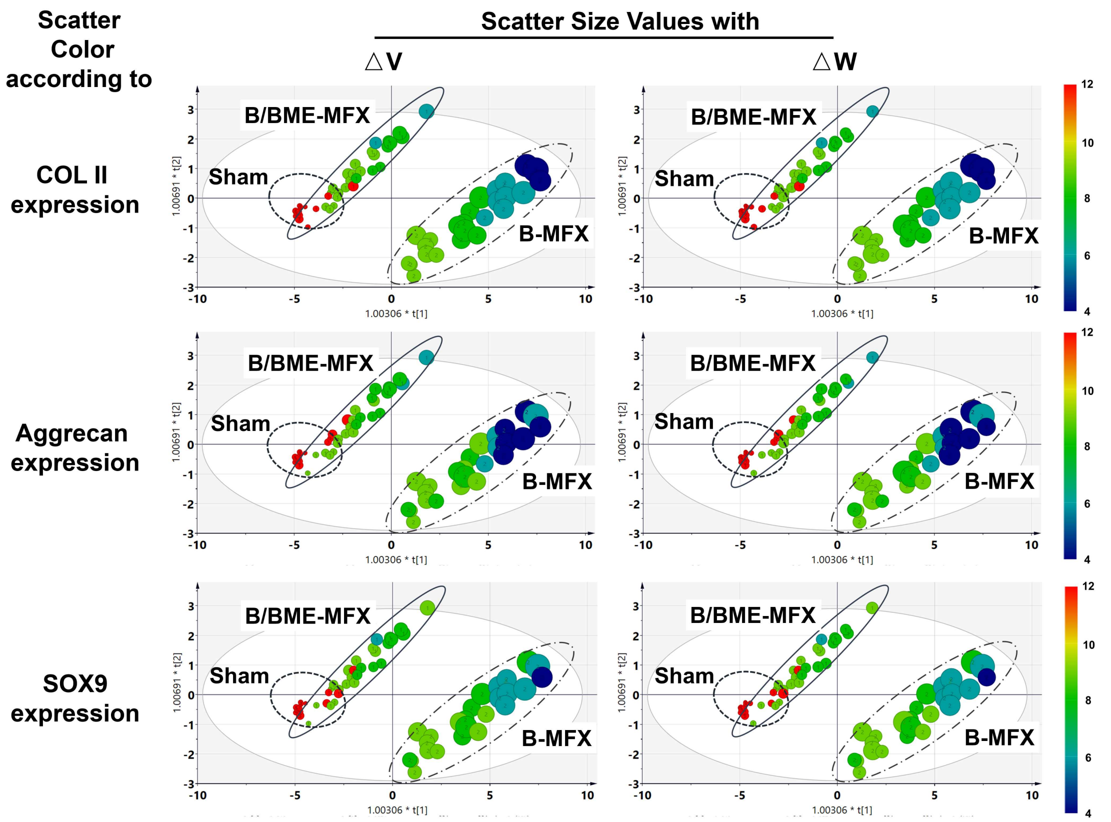
Scatter Color according to
Scatter Size Values with
△V
△W
COL II expression
Aggrecan expression
SOX9 expression
B/BME-MFX
Sham
B-MFX
1.00306 * t[1]
1.00691 * t[2]
12
10
8
6
4

Figure S10

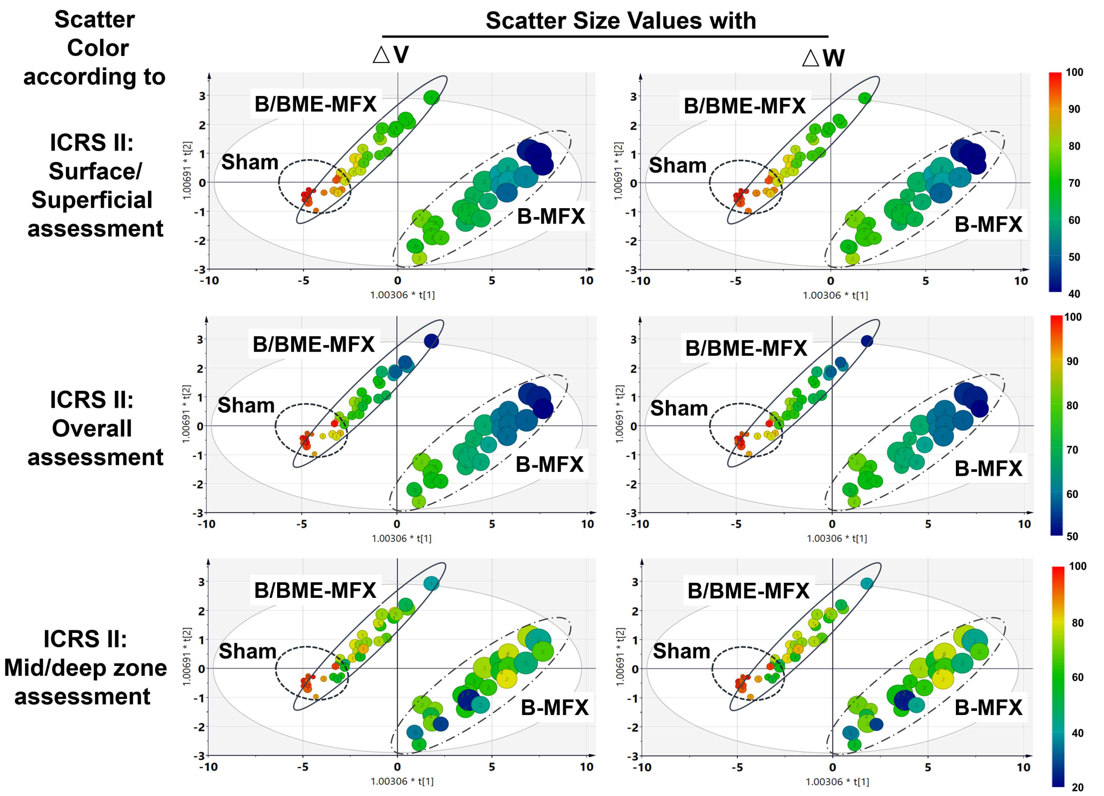
Scatter Color according to
Scatter Size Values with
△V
△W
ICRS II: Surface/ Superficial assessment
ICRS II: Overall assessment
ICRS II: Mid/deep zone assessment
B/BME-MFX
Sham
B-MFX
1.00306 * t[1]
1.00691 * t[2]

Figure S11

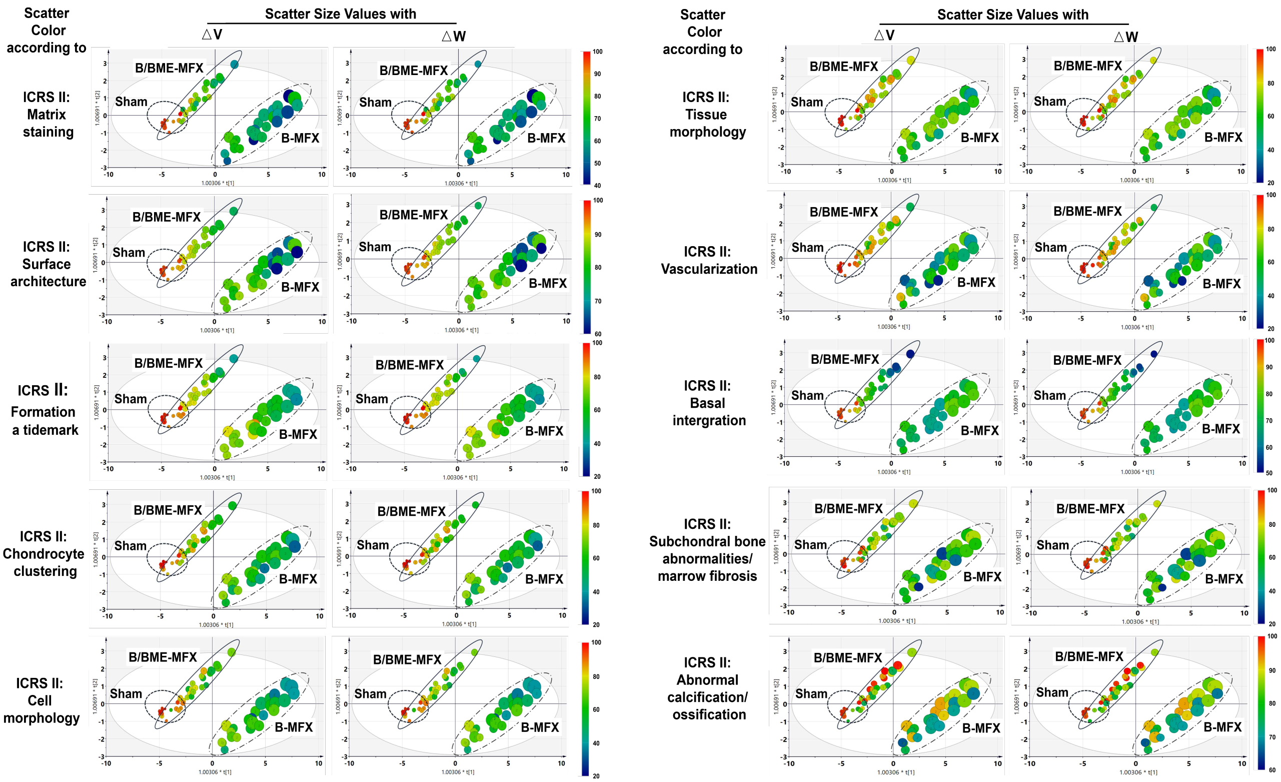
Scatter Color according to
Scatter Size Values with
△V
△W
ICRS II: Matrix staining
ICRS II: Surface architecture
ICRS II: Formation a tidemark
ICRS II: Chondrocyte clustering
ICRS II: Cell morphology
ICRS II: Tissue morphology
ICRS II: Vascularization
ICRS II: Basal intergration
ICRS II: Subchondral bone abnormalities/ marrow fibrosis
ICRS II: Abnormal calcification/ ossification
B/BME-MFX
Sham
B-MFX
1.00306 * t[1]
1.00691 * t[2]

Figure S12

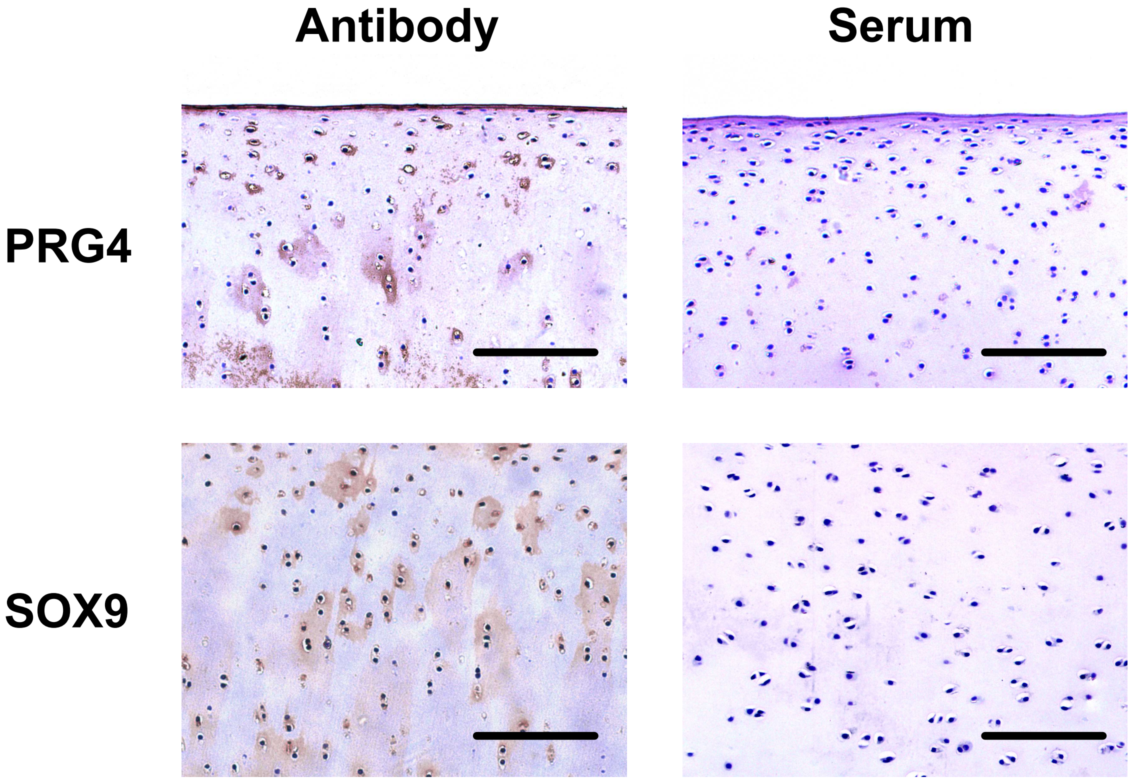

Supplement: Supplementary file 2 — Supplementary figures 7-12. [file thnov10p2538s2.pdf]
